# Relevance of the antibody Fc fragment and epitope valency in protection against malaria sporozoites

**DOI:** 10.1038/s44319-026-00788-3

**Published:** 2026-04-27

**Authors:** Yevel Flores-Garcia, Manuela C Aguirre-Botero, Prabhanshu Tripathi, Emily Locke, Minah Park, Shamika Mathis-Torres, Berenice Salgado-Jimenez, Sonia Herrera-Arevalo, Anisha Gladston, Beejan Asady, Isabelle Coppens, Rogerio Amino, Fidel Zavala

**Affiliations:** 1 Bloomberg School of Public Health. Department of Molecular Microbiology and Immunology. Malaria Research Institute, Baltimore, MD USA; 2 Institut Pasteur, Université Paris Cité, INSERM, Malaria Infection and Immunity, BioSPC 562, ParasitInnov U1347, Paris, France; 3 Vaccine Research Center, National Institute of Allergy and Infectious Diseases, National Institutes of Health, Bethesda, MD USA; 4 Center for Vaccine Innovation and Access, PATH, Washington, DC, USA

**Keywords:** Immunology, Microbiology, Virology & Host Pathogen Interaction

## Abstract

Protection against infection by *Plasmodium falciparum* sporozoites is mediated in part by antibodies that recognize the repeated (NPNA)3 epitopes of the circumsporozoite protein (CSP). To evaluate the role that the antibody Fc region plays in neutralizing sporozoite infectivity, we assess the protective efficacy of Fab fragments obtained from protective human monoclonal antibodies and use antibodies bearing the LALA and KA mutations known to reduce Fc functions. To determine the impact of multiple-antibody binding to CSP on protection, we use sporozoites from genetically modified *P. berghei* strains expressing a CSP containing different numbers of (NPNA)3 epitopes. Our results indicate that Fab fragments and Fc-mutated antibodies have a protective efficacy comparable to intact antibodies, indicating a limited role, if any, of Fc functions in sporozoites in this model system. We also determine that antibody binding to multiple adjacent epitopes and the establishment of homotypic interactions between bound antibodies may not be necessary for protection, as binding to one epitope is sufficient to strongly reduce liver burden while only two separate epitopes are needed to achieve sterile protection.

## Synopsis

Fab fragments of antibodies specific for P. falciparum CSP abolish the infectivity of malaria sporozoites without the involvement of the Fc fragment. These antibodies protect after binding to single epitopes and without establishing homotypic interactions.

- Fab fragments from protective antibodies inhibit malaria sporozoites infection.
- Antibodies lacking Fc functions do not lose their protective efficacy.
- Antibody binding to only 1 or 2 separate epitopes abolishes parasite infection.

## Abstract (web summary)

Fab fragments of antibodies specific for P. falciparum CSP abolish the infectivity of malaria sporozoites without the involvement of the Fc fragment. These antibodies protect after binding to single epitopes and without establishing homotypic interactions.

## Graphical abstract

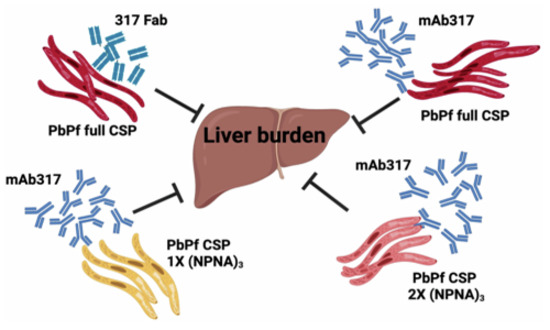

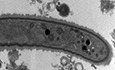

## Introduction

The circumsporozoite protein (CSP) expressed on the surface of *Plasmodium falciparum* sporozoites contains epitopes recognized by antibodies that prevent sporozoite infection and T cells that eliminate or inhibit the development of parasites infecting hepatocytes (Miura et al, 2024). A Phase 3 vaccine trial with RTS,S/AS01, a *P. falciparum* CSP-based malaria vaccine, was performed in Africa in areas with different transmission intensities (RTS, 2015). In children aged 5–17 months, the efficacy against infection began at 74% during the first 3 weeks after the last immunization and waned to 28% at 12 months and 9% at 5 years after vaccination. A booster dose at 18 months increased efficacy to 59%, resulting in 17% efficacy at 5 years. In infants aged 6–12 weeks, efficacy against infection began at 63%, and waned to 11% at 12 months and to 3% at 5 years. A booster dose at 18 months increased efficacy to 58%, resulting in 8% efficacy at 5 years (RTS, 2015; White et al, 2015). Protection conferred by immunization with RTS,S varied in different endemic areas and appeared to depend on the intensity of transmission, as efficacy decreases for children and infants living in regions with moderate to high malaria transmission. Recent studies in Africa indicate that this vaccine reduces death among young children by 13% over 4 years (Wadman, 2023). A second vaccine, R21/Matrix-M, confers comparable efficacy to RTS,S in young African children when compared across similar schedules, endpoints, follow-up duration, and endemicity, although direct head-to-head studies have not been performed. Both vaccines are recommended for use by the World Health Organization and are being introduced across sub-Saharan Africa (Datoo et al, 2024).

The RTS,S and R2 vaccines consist of a portion of the central domain consisting of NPNA repeats and the C-terminal region of the *P. falciparum* CSP. Protection mediated by these vaccines is associated with the levels of circulating antibodies specific for the central repeat domain (White et al, 2015; Kester et al, 2009). Studies using mouse models and transgenic *P. berghei* parasites expressing the *P. falciparum* CSP have shown that passive transfer of vaccine-induced polyclonal or monoclonal antibodies (mAbs) specific for (NPNA)_3_, the epitope of the central repeat domain, immobilizes sporozoites and prevents the in vivo infectivity of transgenic sporozoites expressing the *P. falciparum* CSP (PbPfCSP) (Flores-Garcia et al, 2025; Williams et al, 2024). Gene sequencing from hundreds of *P. falciparum* isolates indicates that the (NPNA)_3_ repeat epitopes are universally conserved in the CSP, and the genetic diversity of this domain is limited to the number of repeats that vary from ~30–50 in different strains (Bahl et al, 2003; Bowman et al, 2013; Zeeshan et al, 2012).

The mechanisms by which anti-CSP antibodies neutralize sporozoite infectivity are still poorly characterized. It is well established that these antibodies inhibit motility of sporozoites injected into the skin (Vanderberg and Frevert, 2004; Flores-Garcia et al, 2018) and induce parasite-dependent cytotoxic effects on sporozoites, causing their death (Aliprandini et al, 2018). Parasite neutralization occurs in the skin (Flores-Garcia et al, 2018) and during migration of sporozoites within the liver parenchyma (Wang et al, 2020), reducing or abolishing sporozoite infection of hepatocytes. In vitro studies suggest the Fc fragments of anti-CSP antibodies may play a role in protection by interacting with cells from the innate immune system that could potentially eliminate or destroy parasites (Suscovich et al, 2020) or by diminishing sporozoite viability through the activation of complement components (Behet et al, 2018). This in vitro effect is also observed when using components and immune sera from human volunteers immunized with RTS,S, and this anti-parasite effect correlates with protection observed after vaccination (Kurtovic et al, 2024). It is however worth noting that other studies have shown that sporozoites activate the complement system via the alternative pathway in the absence of antibodies, and that complement components are deposited in the parasite surface without affecting their in vivo infectivity (Jiao et al, 2024; Kawamoto et al, 1992). Clearly, the protective role of these Fc functions in vivo has yet to be demonstrated.

The protective efficacy of mAbs against the central repeats may be enhanced by the antigen valency present in this CSP domain as the (NPNA)_3_ epitopes are repeated ~10–15 times allowing the binding of several antibodies per CSP molecule. Indeed, in vitro studies have shown that multiple Fab fragments from protective antibodies can bind to the central domain of CSP molecules, establishing homotypic interactions that stabilize and enhance binding to the antigen (Imkeller et al, 2018; Martin et al, 2023). These Fab-Fab interactions have been proposed to be a critical mechanism underlying the protective efficacy of antibodies bound to the CSP.

In this report, we describe research aimed at studying in vivo these two aspects of antibody-mediated protection against sporozoite infection: the role the Fc portion of antibodies may play in neutralization of sporozoite infectivity and the impact of multiple antibody binding on protective efficacy. For these purposes, we used Fab fragments derived from protective human mAbs specific for the central repeat epitopes and sporozoites from several genetically modified transgenic *P. berghei* strains expressing a CSP that contains different numbers of (NPNA)_3_ *P. falciparum* CSP epitopes.

## Results

### Role of IgG Fc portion in protection

**Figure EV1:**
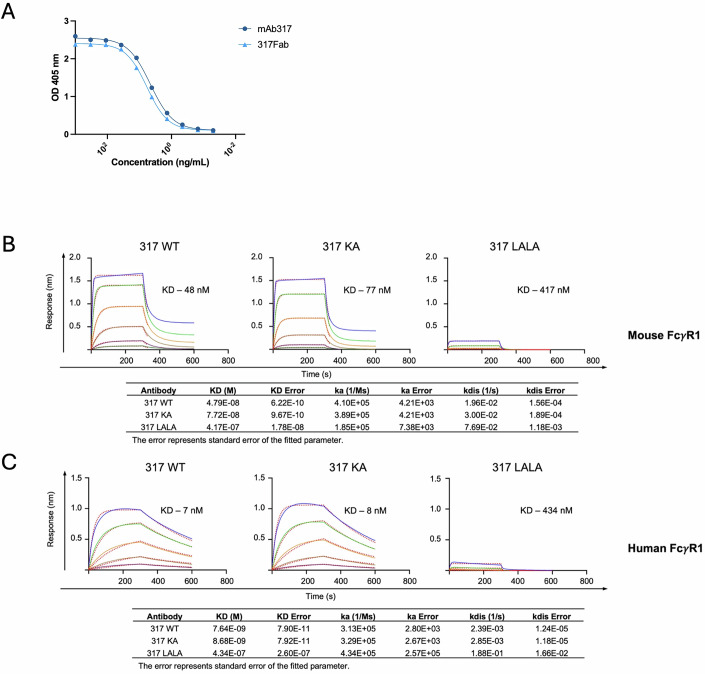
ELISA of mAb317 and 317 Fab binding to CSP and Biolayer interferometry of antibody binding to FcγRI. (**A**) mAb317 (dark blue circles) and 317 Fab (light blue triangles) show the same binding activity to rCSP. ELISA assay using rCSP as antigen was used to evaluate the binding activity of mAb317 and 317 Fab fragments, results of a test run in duplicates. (**B**, **C**) Biolayer interferometry (BLI) analysis of antibody variant binding to mouse and human FcγRI. Binding to mouse (**B**) or human (**C**) FcγRI was assessed by biolayer interferometry (BLI) at physiological pH (7.4). Representative sensorgrams are shown with experimental data overlaid with the global fit (red dotted line) from one experiment run in duplicate. Equilibrium dissociation constants (KD, nM) are indicated on each panel.

We selected the monoclonal antibody 317 (mAb317) to initiate these studies. This antibody, isolated from B-cells of a human volunteer immunized with RTS,S (Oyen et al, 2017), belongs to the IgG VH3-30 antibody family and is specific for the epitope (NPNA)_3_ from the central repeat domain of the *P. falciparum* CSP (Pholcharee et al, 2021). Adoptive transfer of this mAb to mice challenged with sporozoites expressing PfCSP inhibits parasite infection of hepatocytes and confers sterile immunity (Flores-Garcia et al, 2019; Raghunandan et al, 2020). To determine the role that the Fc region of anti-CSP antibodies may have in protection, we compared the protective efficacy of intact mAb and the respective Fab fragments (Fabs) regarding their capacity to neutralize the in vivo infectivity of sporozoites. Fab fragments of mAb317 were generated by papain digestion using a commercially available kit (Pierce ^TM^). Fabs were purified through a protein A column to eliminate partially digested Fc fragments and undigested IgG. The suspension was dialyzed against PBS, and the purity of the Fab preparation was confirmed by SDS-PAGE. ELISA using recombinant CSP as antigen confirmed that these fragments fully retained their antigen-binding capacity (Fig. EV1A).

**Figure 1:**
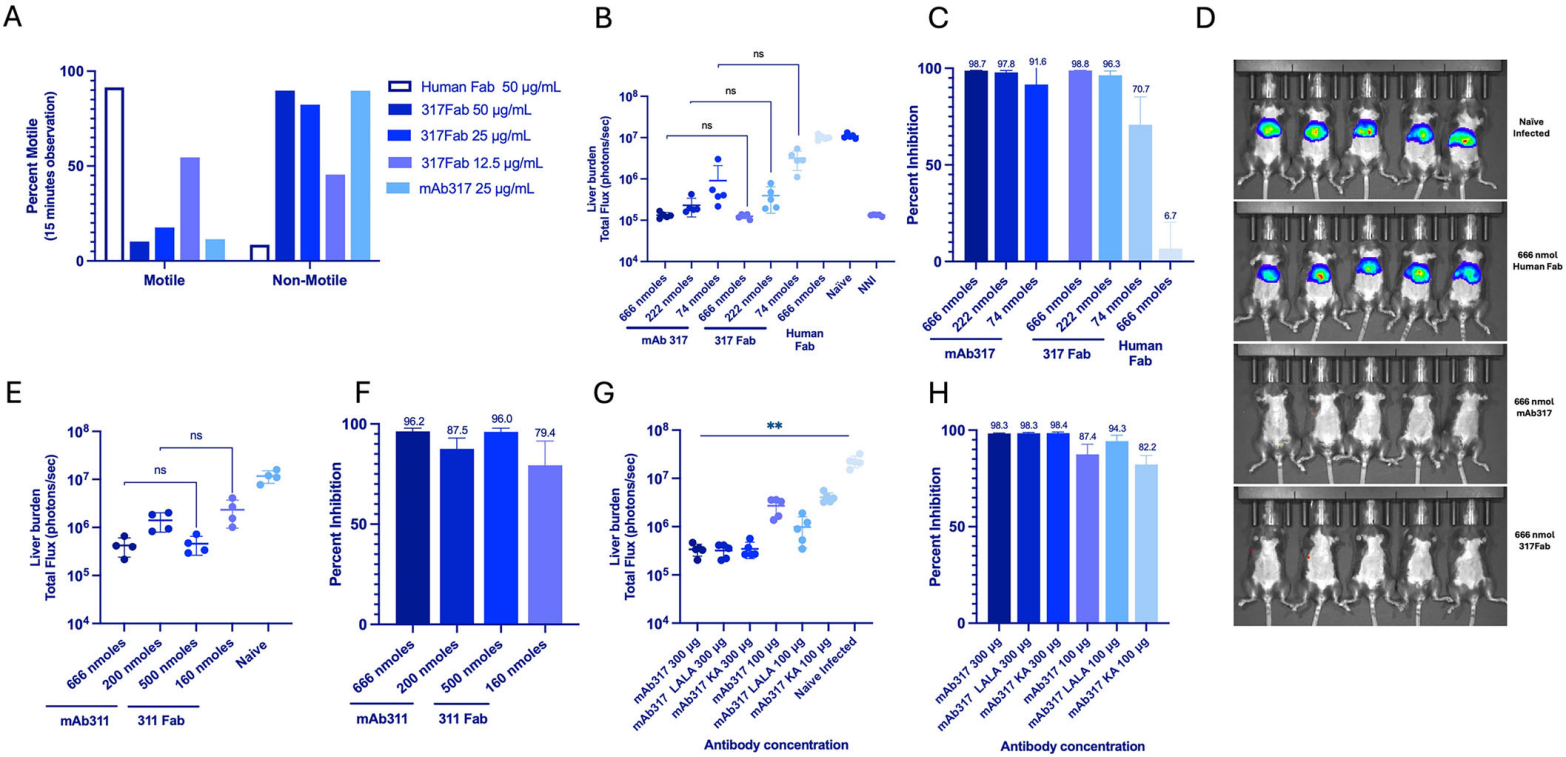
mAb317 IgG and Fab fragments impair in vitro motility and in vivo infectivity of sporozoites, while Fc mutations do not affect the antibody’s protective efficacy. (**A**) Representative graph of three biological replicates showing the effect of mAb317 and 317 Fab on in vitro motility of freshly isolated PbPf full CSP sporozoites. The percentage of sporozoites becoming non-motile after incubation with mAb317 (25 µg/ml) or different doses of 317 Fab was determined using time-lapse microscopy for 15 min at 37 °C. As a control, a non-specific human Fab (50 µg/ml) was used. (**B**) Representative result of three biological replicates, C57Bl/6 mice (5 mice/group) were injected i.v. with equimolar doses of intact mAb317, Fab fragments or control non-specific human Fab and simultaneously challenged with 2000 PbPf CSP sporozoites, the scatterplots show the mean with standard deviation of the parasite liver load, Mann–Whitney *t* test for each dose of mAb or Fab was compared, *P* > 0.05. The histogram on (**C**) shows the parasite burden expressed as percent inhibition from individual mice related to the naive group. (**D**) Representative images of bioluminescence of three biological replicates of mice treated with mAb317 or 317 Fab. (**E**) Representative result of two biological replicates, C57Bl/6 mice (4 mice/group) were injected i.v. simultaneously with different doses of intact mAb311 or Fab fragments and challenged with 2000 PbPf sporozoites. The scatterplots show the mean with standard deviation. Mann–Whitney *t* test for each dose of mAb or Fab was compared, *P* > 0.05. (**F**) Parasite percent reduction in the liver was calculated in individual mice in relation to the naive control group. (**G**) Result of a single experiment where female C57BL/6 mice (5 mice/group) were injected i.v. with 300 µg or 100 µg of mAb317WT or the Fc variants LALA and KA, and challenged 16 h later i.v. with 2000 transgenic PbPf CSP sporozoites. The scatterplots in G show parasite bioluminescence in the liver, mean with standard deviation (total flux, photons/s); Mann–Whitney *t* test for each mAb was compared to liver burden in the naive group, *P* = 0.0079. (**H**) shows the percent reduction, calculated individually in relation to the naive control group. Source data are available online for this figure.

First, we evaluated the effect of IgG and Fabs on the in vitro motility of sporozoites. As shown in (Fig. 1A), after incubation at 37 °C, 317 Fab fragments immobilized parasites in a dose-dependent manner. Control human Fabs have no effect on sporozoite motility.

To compare the in vivo protective efficacy of equimolar amounts of IgG and Fabs, 2000 transgenic *P. berghei* sporozoites expressing the *P. falciparum* CSP and luciferase (PbPf CSP-Luc) (Flores-Garcia et al, 2019) were injected intravenously into mice, together with different doses of intact IgG or Fabs. The simultaneous injection of antibody and parasites was necessary as preliminary experiments indicated that Fabs are swiftly cleared from circulation minutes after intravenous injection. Forty-two hours after sporozoite challenge, the parasite burden in the liver was determined by measuring the parasite’s luciferase activity, as described (Flores-Garcia et al, 2019). As shown in Fig. 1B,C, both IgG and Fabs inhibit sporozoite infection of the liver in a dose-dependent manner and to comparable extents. Passive transfer of control human Fab fragments had no effect on sporozoite infectivity, Fig. 1D. This Fab mediated protective effect is not unique to mAb317 as the same results were obtained with the human mAb311 which belongs to the VH3-33 antibody family and also recognizes preferentially the (NPNA)_3_ epitope. As shown in Fig. 1E, mAb311 IgG and the respective Fabs injected into mice challenged with sporozoites strongly inhibit parasite infectivity, reducing liver burden to a similar extent Fig. 1F.

To further investigate the possible role of the Fc portion in protection, we evaluated the protective efficacy of mAb317 variants containing the LALA (L234A/L235A) and KA (K322A) substitutions in the Fc fragment. These antibodies display the expected binding features to human and mouse FcγR (Fig. EV1B,C). The LALA mutations reduce binding of the Fc portion to Fc gamma RI, RII, and RIII (Shields et al, 2001), strongly reducing ADCC functions, while the KA mutations abolish binding of complement component C1q while minimally affecting binding to FcγR and thus maintaining ADCC functions (Hezareh et al, 2001) (Idusogie et al, 2000). When comparing both the original and the Fc-modified mAb we found that their respective protective efficacy is nearly identical when used at 300 µg (Fig. 1G,H) while mAb317 LALA was slightly more protective when used at 100 µg. Taken together, these results indicate that the neutralization of sporozoite infectivity by this protective mAb may not depend on Fc gamma receptor binding functions or complement activation.

**Figure 2:**
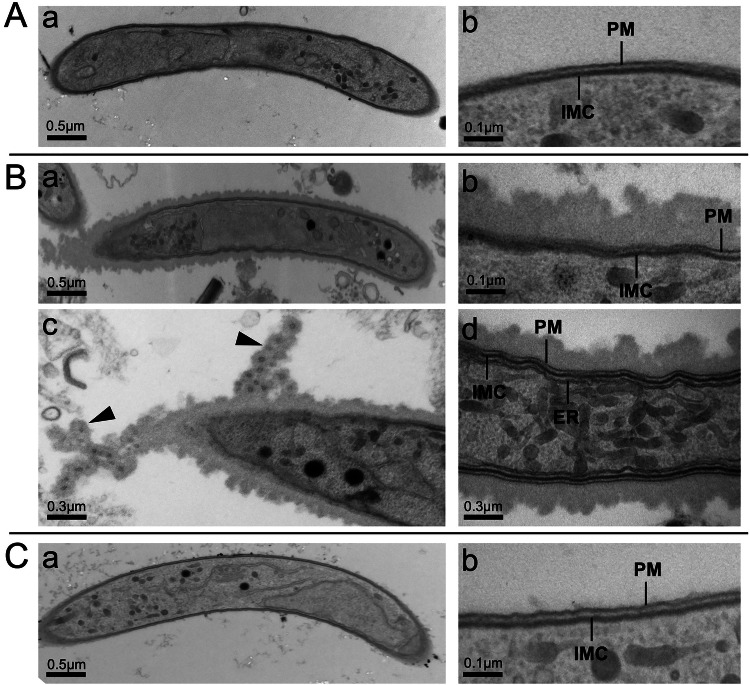
Ultrastructure of PbPf CSP sporozoites exposed to mAb317 IgG. EM of PbPf CSP sporozoites incubated with mAb Isotype IgG control (**A**), mAb317 anti-(NPNA)_3_ (**B**) or 317 Fab anti-(NPNA)_3_ (**C**). For each condition, sporozoites were incubated 15 min with the antibodies at 75 µg/ml at 37 °C before fixation and processing for EM. In contrast to sporozoites shown in (**A**, **C**) (a, b), sporozoites incubated with full-length mAb317 exhibit a large fluffy coat beyond the parasite plasma membrane (PM; a, b). Arrowheads in (c) illustrate filaments made of shed coat protruding from the parasite body (c). Panel d shows thin ER elements reorganized beneath the inner membrane complex (IMC). Scale bars = a, 0.5 µm; b, 0.1 µm; and 0.3 µm for (c, d) as indicated. Source data are available online for this figure.

It is worth noting that Fabs do not induce shedding of the sporozoite surface antigen, i.e., the circumsporozoite precipitation reaction (CSPR), which is commonly observed when incubating parasites with anti-sporozoite antibodies (Cochrane et al, 1976). We conducted transmission electron microscopy (EM) studies to inspect potential alterations of the sporozoite ultrastructure following incubation of extracellular parasites with mAb317, in comparison with sporozoites exposed to 317 Fab or a mAb isotype IgG control (Fig. 2A–C). After 15 min exposure with either antibody prior to fixation, sporozoites incubated with full-length mAb317 exhibited a large fluffy coat, up to 0.4 micron in thickness, covering the parasite surface (Fig. 2B). In contrast, sporozoites exposed to the 317 Fab did not exhibit any change at the plasma membrane (Fig. 2C), similar to parasites incubated with an irrelevant antibody (Fig. 2A). It is likely that the coat surrounding the parasite exposed to full-length mAb317 is made of aggregates of anti-CSP antibodies and CSP that are shed from the plasma membrane, as described in early studies for the antibody-induced CSPR (Cochrane et al, 1976). We also noticed long filaments extended from the coat that contained small vesicles, suggesting shedding of CSP material from the plasma membrane by vesiculation (Fig. 2B,c). Another observation was the remodeling of the endoplasmic reticulum into narrow tubules and their relocation beneath the inner membrane complex, which could be consequential to an external stress exerted by antibody damage (Fig. 2B,d).

**Figure 3:**
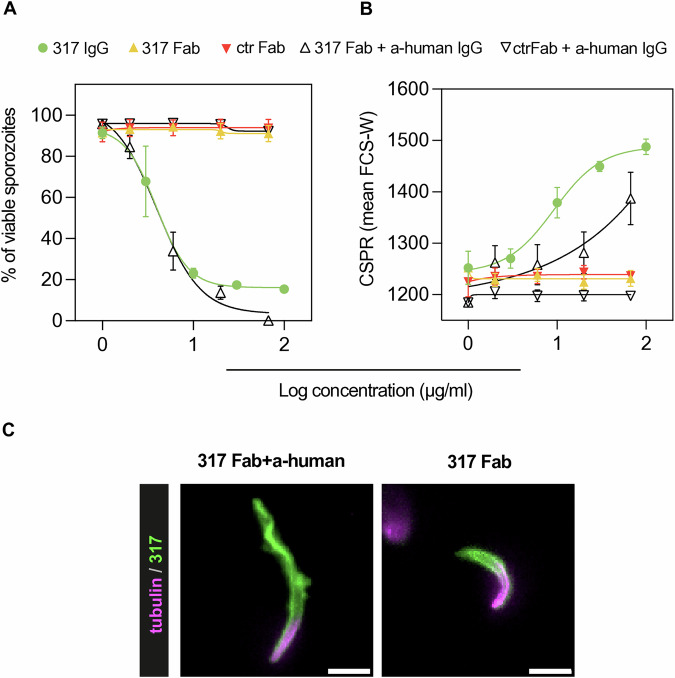
Monovalent 317 Fab does not kill sporozoites in suspension. (**A**) In vitro lethality and (**B**) Circumsporozoite precipitation reaction (CSPR) of mAb317 (green, circle), 317 Fab (yellow, triangle), and a control Fab (ctr Fab, red, reverse triangle) induced in PbPf sporozoites after 45 min of incubation at 37 °C measured by cytometry. In addition to the 317 Fab, 150 µg/ml of a donkey anti-human IgG antibody Alexa 647 was added simultaneously (transparent, triangle). *n* = 3 biological replicates, data are presented as mean ± SEM. (**C**) Representative images of a sporozoite after incubation with 317 Fab with or without anti-human antibody 647. Samples with only 317 Fab were labeled with the same anti-human after fixation. Scale bar = 5 µm. Source data are available online for this figure.

Finally, we evaluated the parasite-dependent cytotoxic effect of these antibodies on sporozoites, a mechanism known to mediate the neutralization of parasite infectivity (Aliprandini et al, 2018). Incubation of 317 Fab with sporozoites in suspension for 45 min at 37 °C has shown that this monovalent binding fragment is neither killing parasites (Fig. 3A, orange triangle) nor shedding the CSP (Fig. 3B,C, orange triangle), unlike the bivalently mAb317 (Fig. 3A,B, green circle). However, the cross-linking of Fabs using an anti-human IgG mAb restored both activities (Fig. 3A–C, open triangle), showing that the killing and CSPR activities likely depend on the cross-linking of adjacent CSP molecules on the sporozoite surface. Taken together, these results indicate that the antibody-induced CSP shedding and the killing of sporozoites activated in suspension are not required for sporozoite neutralization after intravenous (i.v.) inoculation.

### Epitope valency and protective efficacy of mAbs

Cryo-EM studies using human mAbs specific for the epitope (NPNA)_3_ have shown that multiple Fab fragments can bind to a single CSP molecule and establish homotypic interactions. These Fab-Fab interactions help stabilize antibody binding and it is believed to be an important feature underlying the protective efficacy of these antibodies (Imkeller et al, 2018), (Martin et al, 2023).

**Figure 4:**
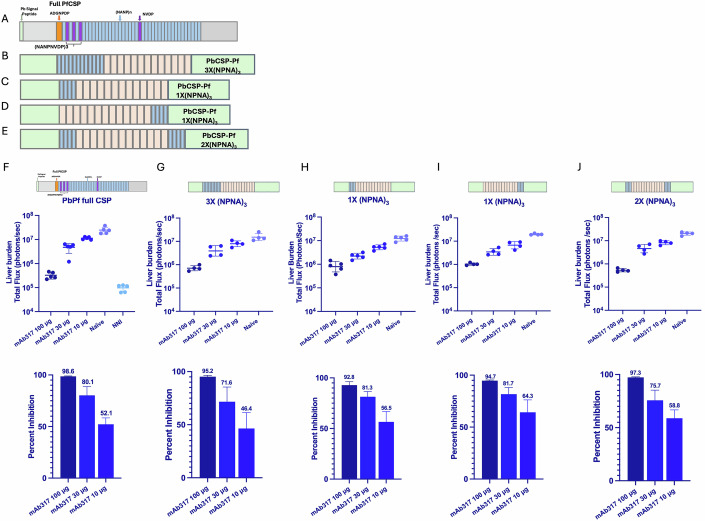
Schematic representation of CSP versions expressed by different Pb transgenic parasite lines. mAb317 efficiently inhibits the infectivity of sporozoites expressing different numbers of (NPNA)3 epitopes. (**A**) *P. berghei* (Pb) sporozoites expressing full-length PfCSP, PfCSP N- and C-termini (gray), junction region ADGNPDP (JR, orange), 4 NVDP minor repeats (MR, purple), and 38 central major repeats (CR, blue) are indicated. (**B**) *P. berghei* CSP expressing 3× (NPNA)_3_ from PfCSP. (**C**) *P. berghei* CSP expressing 1× (NPNA)_3_ from 5’ PfCSP. (**D**) *P. berghei* CSP expressing 1× (NPNA)_3_ from 3’ PfCSP. (**E**) *P. berghei* CSP expressing 2× (NPNA)_3_ from PfCSP one at 5’ and the second one at 3’end of the repeat domain. Representative results of two biological replicates, C57Bl/6 mice (4–5 mice per group) were passively immunized with different doses of mAb317, 100 µg, 30 µg, or 10 µg, and 6 h later challenged intravenously with 2000 transgenic sporozoites expressing (**F**) PbPf full CSP, (**G**) 3× (NPNA)_3_, (**H**) 1× (NPNA)_3_-5’, (**I**) 1× (NPNA)_3_-3’, or (**J**) 2× (NPNA)_3_. The scatterplots show mean +/− standard deviation. Two-way ANOVA, Kruskal–Wallis test showed no statistical difference in percent inhibition between different groups. *P* = 0.9422. The percent reduction was calculated in relation to the naive control mice. Source data are available online for this figure.

To determine whether the binding of multiple antibodies to the repeat domain is in fact a critical requirement for in vivo neutralization of sporozoite infection, we developed *P. berghei* parasite lines expressing modified CSP molecules that contain different numbers of (NPNA)_3_ epitopes (Fig. 4A–E, Reagents and Tools table). We generated a parasite line in which the entire repeat domain of the endogenous *P. berghei* CSP was replaced by a *Pf*CSP insert consisting of 38 NANP repeats containing 11X(NPNA)_3_ epitopes (Fig. 4A). Other parasite lines express 12 NANP repeats containing 3X(NPNA)_3_ epitopes (Fig. 4B), or 4 NANP repeats containing a single (NPNA)_3_ epitope located at the 5’ or 3’ ends of the repeat domain, respectively (Fig. 4C,D). Finally, we generated an additional parasite line containing two 4 NANP repeats; one of them located at the beginning of 5’ repeat domain, and the second one at the 3’ end of the repeat domain, each containing a single (NPNA)_3_ epitope, separated by 104 amino acid residues of the *P. berghei* CSP repeat domain (Fig. 4E).

**Figure EV2:**
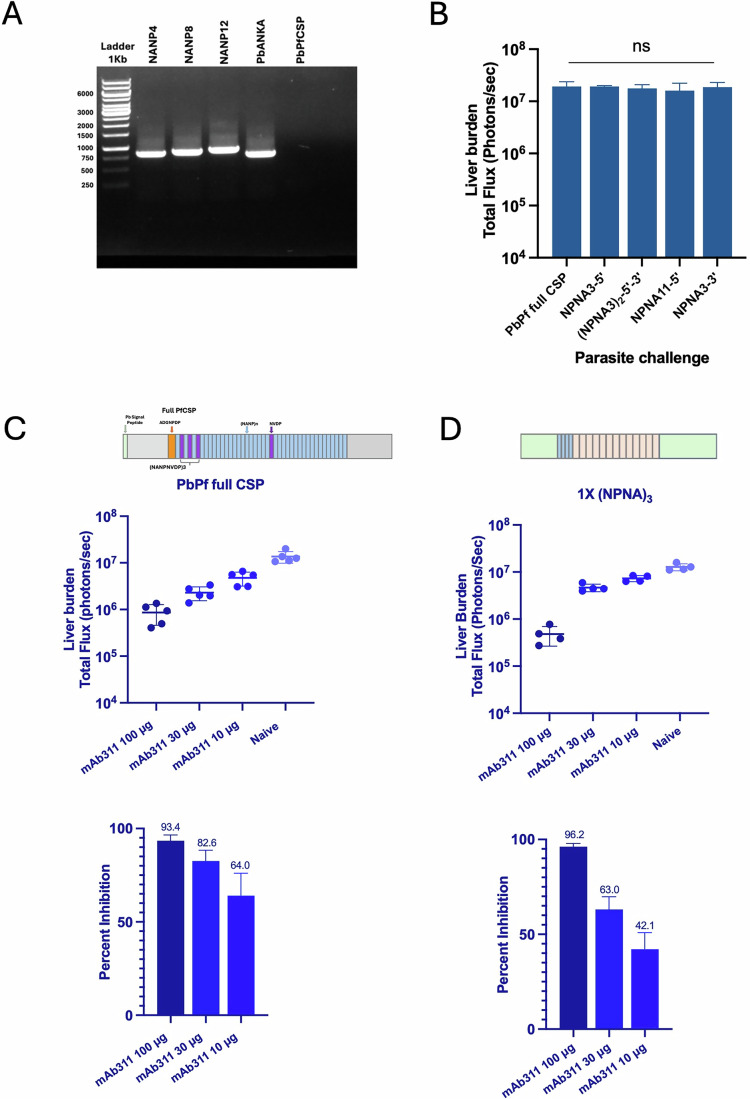
Characterization of the transgenic parasite lines regarding the gene expression and infectivity. (**A**) The new transgenic sporozoite lines show a size shift in the modified CSP gene and (**B**) have comparable infectivity while expressing different numbers of NANP repeats. (**C**) Anti-CSP mAb311 confers protection when the parasites express 1 or 11 (NPNA)_3_ epitope (**A**) PCR products showing different sizes of the modified CSP gene from DNA isolated from the new transgenic parasites. (**B**) Female C57BL/6 mice (5 mice/group) were injected i.v. with 2000 transgenic sporozoites. The graph shows parasite bioluminescence in the liver expressed by the transgenic parasites, mean with standard deviation. Two-way ANOVA showed no statistical differences in infectivity among parasite lines. C57BL/6 mice (*n* = 4–5) were injected i.v. with 100, 30, or 10 μg of mAb311 and i.v. challenged 6 h later with 2000 transgenic sporozoites expressing (**C**) PbPf full CSP, (**D**) 1×(NPNA)_3_. Upper panels: parasite burden in the liver (total flux, photons/s); bottom panels: percent inhibition in liver burden mediated by mAb311. The scatterplots show the mean and standard deviation of one of two biological replicates.

First, we established that the new transgenic parasite lines have comparable in vivo infectivity. All parasite lines generated in this study express luciferase, which enables the monitoring of their infective capacity in mice by measuring bioluminescence in the liver using an IVIS imager. Mice were injected intravenously with 2000 sporozoites from the different parasite lines, and the liver burden was determined 42 h later by measuring luciferase activity (Fig. EV2B). The results indicate that all parasite lines have comparable infectivity, and the addition of NPNA sequences to the endogenous *P. berghei* CSP does not affect the infectivity of the sporozoites.

To determine the capacity of antibodies to neutralize sporozoite infection, we used mAb317 specific for the (NPNA)_3_ epitope (Oyen et al, 2017). This antibody does not affect the infectivity of wild-type *P. berghei* sporozoites or transgenic parasites expressing *P. falciparum* junction epitope and only minimally affects sporozoites expressing minor repeat epitopes (Flores-Garcia et al, 2021). Mice were injected intravenously with 100, 30, or 10 µg of mAb317, and 6 h later they were challenged with the respective transgenic sporozoites. As seen in Fig. 4F, mAb317 strongly inhibited infection of sporozoites expressing the entire *P. falciparum* CSP. Next, we evaluated the protective efficacy of this antibody against sporozoites expressing a CSP with fewer (NPNA)_3_ epitopes. As shown in Fig. 4G, this mAb also efficiently neutralizes the infectivity of sporozoites expressing 3× (NPNA)_3_ epitopes. Moreover, as shown in Fig. 4H–J, the degree of protection mediated by mAb317 against an intravenous sporozoite challenge using parasites expressing 2× or 1× (NPNA)_3_ epitopes is entirely comparable to the inhibition observed against parasites expressing the entire PfCSP. This is surprising as the antibody binding to each CSP expressing a single (NPNA)_3_ is most likely monovalent, while multiple and/or bivalency antibody binding, i.e., is likely to occur when the CSP expresses 11× or 3× (NPNA)_3_ epitopes. The location of the (NPNA)_3_ epitope within the CSP does not appear to be important as the neutralization of parasites expressing 1X(NPNA)_3_ at the 3’ end of the repeat domain was comparable to the inhibition of parasites expressing 1× (NPNA)_3_ at the 5’ end. There were no statistically significant differences amongst these groups (two-way ANOVA, Kruskal–Wallis test). This pattern of neutralizing activity of mAb317 against parasites expressing different numbers of (NPNA)_3_ is also observed when using mAb311, an *IGHV3:33* encoded antibody that also binds this epitope (Fig. EV2C,D).

**Figure 5:**
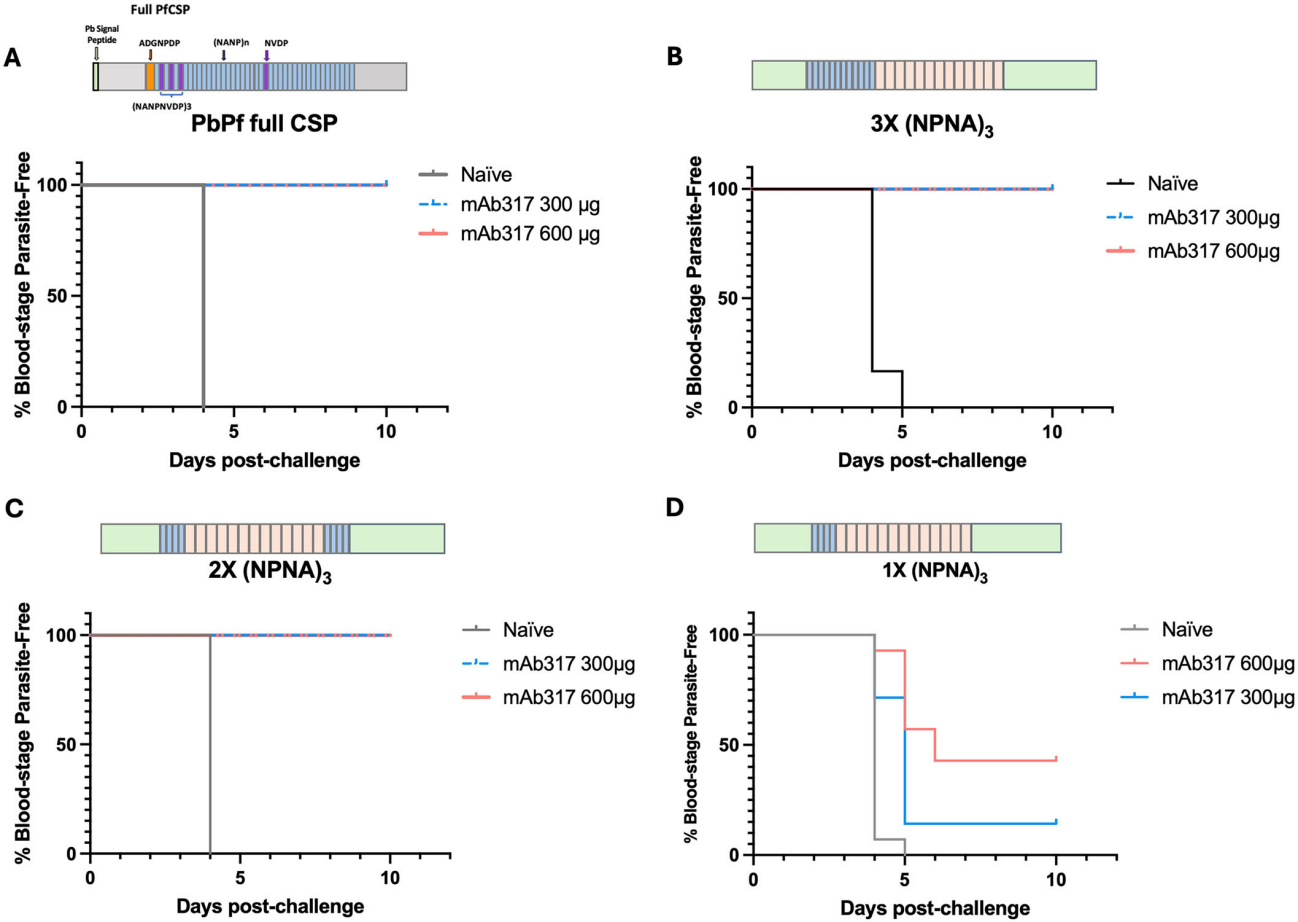
Passive transfer of mAb317 confers sterile protection in mice exposed to the bites of mosquitoes infected with different transgenic parasite lines. Kaplan–Meier survival curves of C57BL/6 mice (total of 14 mice; combined from two biological replicates) receiving mAb317 (600 µg or 300 µg) and challenged 6 h later via the bites of 5 mosquitoes infected with SPZ expressing (**A**) PbPf full CSP, (**B**) 3× (NPNA)_3_, (**C**) 2× (NPNA)_3_, (**D**) 1× (NPNA)_3_. Source data are available online for this figure.

Next, we determined the capacity of mAb317 to confer sterile immunity to mice challenged by exposure to the bites of mosquitoes infected with the different sporozoite lines (Flores-Garcia et al, 2019). Protection was determined by assessing the appearance of parasite-infected erythrocytes from days 4 to 10 after challenge. Passive transfer of 600 or 300 µg of mAb317 protects 100% of the mice (Fig. 5A). Similarly, we observed 100% sterile protection in mice receiving 600 or 300 µg of antibody and challenged with parasites expressing 3 or 2 (NPNA)_3_ epitopes (Fig. 5B,C). Mice challenged with parasites expressing a single (NPNA)_3_ were partially protected when receiving 600 µg of mAb317, and only 15% sterile protection was observed in mice receiving 300 µg (Fig. 5D).

Taken together, these results indicate that IgG binding to CSP expressing a single (NPNA)_3_ epitope can inhibit the infectivity of most sporozoites injected intravenously. The binding to 2 separate epitopes enhances this protection, when mice are challenged by mosquito bites, to an extent comparable to that observed with parasites expressing the entire PfCSP. These results indicate that antibody binding to a single epitope can successfully neutralize sporozoite infectivity, suggesting that multiple/bivalency antibody binding to the CSP and the formation of homotypic interactions between antibodies may have a limited impact on the ability of antibodies to neutralize sporozoite infectivity in the liver. However, it is evident that the presence of two separate epitopes enhances the antibody protective efficacy as the proportion of sterile protection in mice challenged through the bites of infected mosquitoes is significantly increased (Fig. 5C,D).

**Figure 6:**
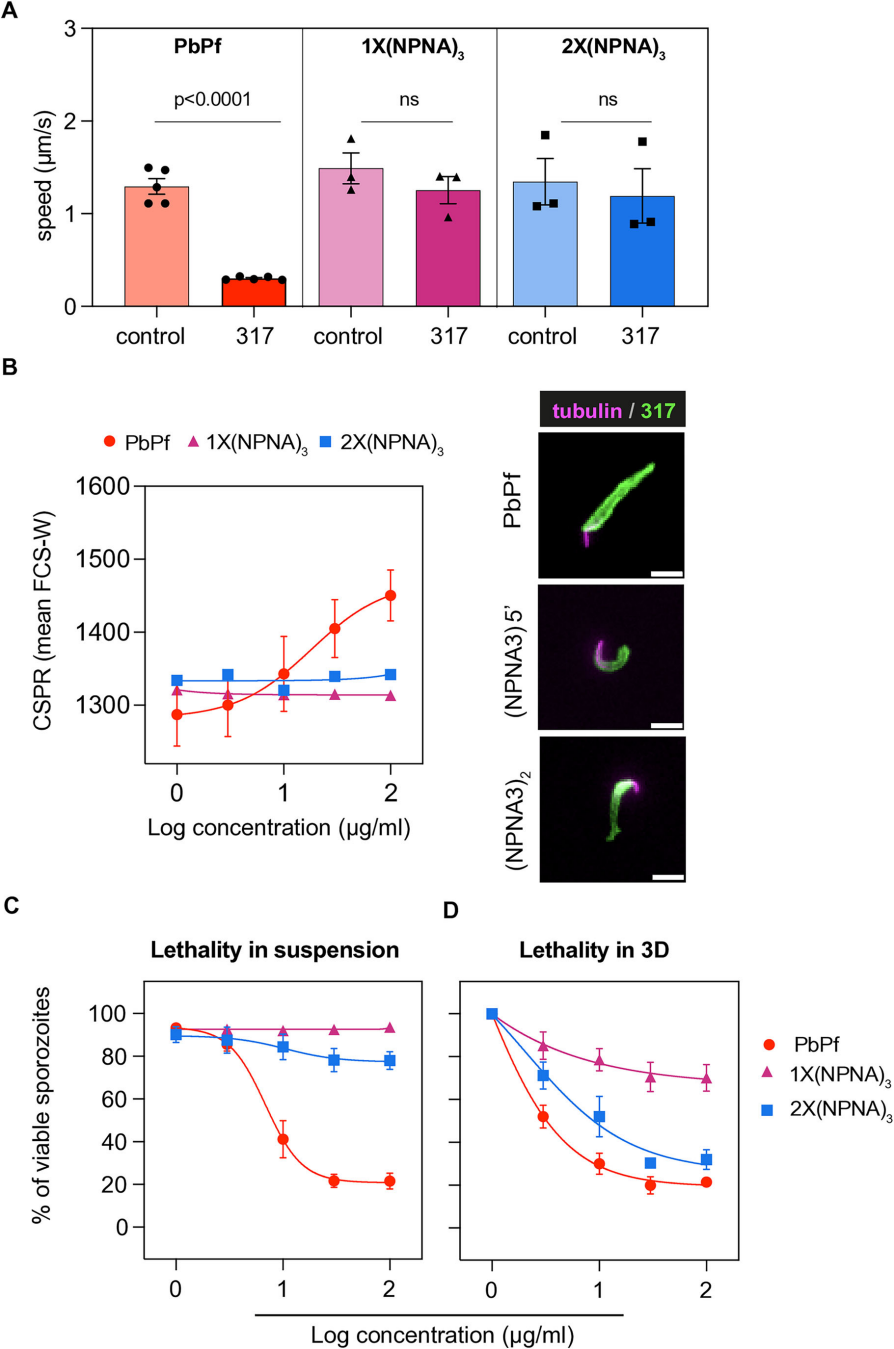
The cytotoxic effect of mAb317 depends on the different numbers of (NPNA)_3_ epitopes. (**A**) The average speed of PbPf CSP, 1× (NPNA)_3_ and 2X(NPNA)_3_ sporozoites after 3 min incubation in 10% FCS/PBS in the absence (control) or presence of 10 µg/ml mAb317, *P* < 0.0001, *P *= 0.1816, *P* = 0.9727, respectively. (**B**) Left: Estimated CSPR length after incubation with mAb317 as analyzed by flow cytometry. Right: Representative images of sporozoites of the respective lines after incubation with 100 µg/mL mAb317. The apical end of the parasite was revealed using SPY tubulin. Scale bar: 5 µm. (**C**) Lethality of 317 IgG on the different strains in suspension or (**D**) in 80% 3D-Matrigel after 45 min incubation at 37 °C, (*n* = 4), (**A**–**C**) *n* = 3 biological replicates. Graphs show mean with standard error. Source data are available online for this figure.

Finally, we characterized the effect of the in vitro activity of mAb317 on some of these sporozoite lines using the PbPf CSP parasite as a positive control. As shown in Fig. 6, the effect of mAb317 on CSPR, motility inhibition, and killing of sporozoites in suspension was very similar in the parasite lines expressing 1 and 2 epitopes (Fig. 6A–C). In contrast, mAb317 strongly reduces the motility of PbPf CSP sporozoites (Fig. 6A). The killing of sporozoites by mAb317 was assessed using a 3D assay, which measures the antibody-mediated cytotoxicity on sporozoites embedded in Matrigel, and has been shown to best correlate with in vivo protection after mosquito bite challenge (Aguirre-Botero et al, 2023). Using this assay, we found that mAb317 displayed a dose-dependent cytotoxic activity on sporozoites expressing 2X(NPNA)_3_ epitopes, similar to that observed with sporozoites expressing the entire PfCSP. Both parasite lines were more susceptible to mAb317 3D killing than parasites expressing only 1X(NPNA)_3_ 5’ (Fig. 6D). This likely explains the higher protection elicited by mAb317 in passively immunized mice observed after challenge with sporozoites expressing ≥2× (NPNA)_3_ epitopes (Fig. 5), through the bites of infected mosquitoes.

## Discussion

The Fc portion of immunoglobulin plays important roles mediating the anti-microbial effector functions of antibodies by activating components of the complement system and triggering the anti-microbial activity of cells from the innate immune system (Junker et al, 2020). The Fc fragment is also critical to ensure prolonged circulation of antibodies in blood through its interactions with FcRn receptors (Dall’Acqua et al, 2006). Several studies in animal models (Flores-Garcia et al, 2025; Oyen et al, 2017) and humans (Gaudinski et al, 2021; Wu et al, 2022) have conclusively demonstrated that antibodies against the *P. falciparum* CSP play a major role in protection. In vitro studies suggest that the Fc fragment of these antibodies may activate complement components enhancing the neutralizing activity of antibodies (Kurtovic et al, 2024) and promote the activation of NK cells or neutrophils that might destroy sporozoites before invasion of hepatocytes (Suscovich et al, 2020), (Feng et al, 2021). However, the notion that these Fc-mediated functions are necessary for in vivo protection has yet to be demonstrated.

The studies described in this report, using protective mAbs, indicate that Fab fragments obtained after enzymatic digestion of the Fc portion strongly neutralize parasite infectivity, and the magnitude of this inhibitory activity is comparable to that observed with intact antibodies. Moreover, genetically modified mAb317 bearing the LALA and KA mutations in the Fc fragment, which reduce binding of the Fc portion to Fc gamma RI, RII, and RIII and binding of complement component C1q, respectively, do not reduce the protective effect of this mAb. These findings are consistent with earlier studies in mice using *P. yoelii* sporozoites, indicating that depletion of neutrophils (Aliprandini et al, 2018; Mac-Daniel et al, 2014), monocytes, and the absence of complement components or FcγR signaling (Aliprandini et al, 2018) do not reduce the protective efficacy of the anti-sporozoite antibodies. Moreover, they are in agreement with early studies with *P. berghei* sporozoites, indicating that Fabs of an anti-CSP mAb inhibit sporozoite infection in mice (Potocnjak et al, 1980). These findings may not be surprising as infection of hepatocytes can occur as soon as 2–15 min after sporozoites reach the bloodstream (Shin et al, 1982; Sidjanski and Vanderberg, 1997) and, therefore, antibody-mediated mechanisms involving the activation of other cell types may not be effective. Recent studies have analyzed Fc-dependent functions of anti-CSP antibodies bearing mutations on the Fc portion, including anti-central repeat, anti-junction, and anti-minor repeat antibodies. In vitro assays to evaluate ADCC functions using synthetic constructs or sporozoites, indicate that Fc-mutated antibodies had in general a decreased in vitro activity (Stefanutti et al, 2025). This study also included in vivo experiments, which indicated that none of these mutated antibodies have a decreased protective efficacy when evaluating parasite infection by measuring liver burden, although it is claimed that some of the mutated antibodies may have less efficacy when measuring the occurrence of blood parasitemia. In contrast, our results are in agreement with studies, indicating that antibodies against junction and minor epitopes carrying the LALA mutation do not have a decreased protective efficacy (Kisalu et al, 2025; Wang et al, 2024). It is conceivable that in humans some of these Fc effector functions may be operating, as suggested by the in vitro studies that indicate a correlation of these Fc functions and protection. However, caution must be exercised before reaching conclusions based on in vitro assays, as the effector functions that are being measured are significantly influenced by the antibody avidity to antigens (Oostindie et al, 2022), which by itself is an important determinant of in vivo protection. As recent studies have shown that the passive transfer of mAbs against CSP to humans protects against sporozoite infection, it may be possible to directly answer these questions in human trials.

Importantly, however, while the Fc portion may not be critical for a direct anti-parasite effect, Fc functions influence protective immunity through other mechanisms. As shown for anti-CSP mAbs, the LS modifications of the Fc fragment increase the avidity for the FcRn, allowing longer-lasting circulation in blood and thus provide a critical improvement for the development of mAbs to be used for prophylaxis, in human trials (Kisalu et al, 2021). In addition, as shown in other systems, the Fc–Fc gamma receptor interactions can influence the development of adaptive immune responses by modulating T cell functions, as well as antigen processing and presentation (Junker et al, 2020). Finally, there are critical protective activities mediated by antibodies that depend on their transference from peripheral blood to tissue parenchyma, as is the case for the neutralization of malaria sporozoites in the skin. The mechanisms that regulate this important function and the likely role of the Fc fragment remain poorly characterized. Another important feature regulating Fc functions of human immunoglobulin is the N-glycosylation present at conserved Asparagine 297 in the CH2 domain. The Fc glycosylation of the Fc is closely associated with antibody-dependent cell cytotoxicity (ADCC), a critical component of immune responses against pathogens and cancer cells. Afucosylated antibodies, with low fucose content, have an increased affinity for FcgR and exhibit enhanced ADCC activity. In ADCC, immune cells like natural killer cells (NK) are activated when they encounter target cells bound by antibodies. Afucosylated antibodies are more effective at recruiting and activating NK cells, leading to more potent target cell destruction. It is also believed that afucosylated antibodies increase phagocytosis in macrophages and increase ADCC activity of gd T cells and PMN (Golay et al, 2022). The potential role of Fc glycosylation in antibody effector functions against sporozoites is an aspect that has yet to be studied.

Unlike intact IgG, the protective Fab fragments do not induce shedding of CSP, indicating that this reaction is not a requisite for sporozoite neutralization. However, both IgG and Fabs have the capacity to decrease sporozoite motility, a mechanism by which antibodies could inhibit sporozoite infection. Fabs do not kill sporozoites in suspension, but this is achieved by cross-linking Fabs on the parasite surface using a secondary anti-IgG antibody, thus indicating that antibody bivalence is essential for lethality. Using Fab2 fragments, we attempted to determine whether these antibody fragments could cross-link two neighboring CSP molecules and increase their anti-parasite activity. Unfortunately, the enzymatic treatment to obtain these fragments severely affected the antibody’s ability to bind the CSP.

In vitro studies have shown that multiple Fab fragments bind to a single CSP molecule and establish homotypic interactions that stabilize antigen-antibody complexes and may enhance the protective efficacy of these antibodies (Imkeller et al, 2018; Martin et al, 2023). The central repeat domain in *P. falciparum* consists of approximately 30–50 tandem NANP repeats and therefore several antibodies—perhaps as many as 5–6 IgGs—can potentially bind simultaneously to this region. Structural studies show that multiple Fab’s bind to recombinant CSP and form a helical structure stabilized by homotypic interactions (Oyen et al, 2018). This unique feature raises the question of whether binding multiple antibodies per molecule is critical for protection. To determine the relevance of antigen valency of the CSP for in vivo protection, we generated several transgenic *P. berghei* strains expressing a modified CSP molecule that contains different numbers of (NPNA)_3_ epitopes. When comparing the protective efficacy of the mAbs against these different parasites by measuring parasite liver burden after intravenous inoculation of sporozoites, we found that neutralization was equally efficient against sporozoites expressing a CSP with 1X, 2X, 3X (NPNA)_3_ epitopes or the entire PfCSP with nearly a dozen epitopes. However, when assessing sterile immunity after exposing mice to the bites of infected mosquitoes, we found that parasites expressing a single (NPNA)_3_ epitope are less sensitive to the effect of antibodies. This result aligns with the lower killing of this parasite line by the anti-PfCSP antibody in the 3D cytotoxicity assay, known to correlate with protection after mosquito bite challenge (Aguirre-Botero et al, 2023). Our data also show that expression of two separate epitopes renders parasites highly susceptible to antibody killing activity in the 3D matrix, and thus efficiently neutralized, when delivered by infected mosquitoes in the skin. This result also indicates that antibody killing activity is associated with the CSP cross-linking mediated by bivalent effectors binding to repeated epitopes of adjacent molecules. The present studies have focused on antibodies to (NPNA)_3_ repeats because they are known to be protective and are induced by the current sporozoite vaccines RTS,S and R21 (Oyen et al, 2017; White et al, 2015). Monoclonal antibodies obtained from sporozoite-immunized volunteers that are specific for the Junction region (CIS43) (Kisalu et al, 2018) and minor repeat epitope (L9) (Wang et al, 2020) are also protective and have similar features to mAbs specific for the central repeat. Modified versions of these antibodies carrying the LALA mutation do not show a decreased protective efficacy. Genetic modifications of the Fc portion to increase the affinity for the Fc receptor do not affect the protective efficacy of L9 mAb, while a modest increase was observed in CIS43 mAb, although the reason for this increased efficiency remains to be defined (Kisalu et al, 2025). CIS43 and L9 antibodies are unlikely to generate homotypic interactions as they are protective against parasites expressing a single epitope within the CSP. This is similar to the effect of mAb317 against parasites expressing a single NPNA_3_ epitope (Flores-Garcia et al, 2025; Flores-Garcia et al, 2021). mAbs anti C-term have shown a modest degree of efficacy (Beutler et al, 2022), although anti C-term induced by RTS,S are not protective (Flores-Garcia et al, 2025). These antibodies recognize single epitopes and are unlikely to establish homotypic interactions. It is not known if they activate Fc functions.

Taken together, these results indicate that antibody binding to a CSP expressing 1X or 2X (NPNA)_3_ epitopes can efficiently inhibit the infectivity of most sporozoites. These results suggest that multiple antibody binding to the CSP and the formation of homotypic interactions between antibodies may have a limited impact on the protective efficacy of these antibodies. While the monovalent binding of effectors to CSP is enough to neutralize sporozoites during liver infection, binding of bivalent IgG to two distant epitopes of the CSP on the parasite surface leads to a 3D cytotoxic death in vitro and an efficient neutralization of sporozoites during their passage through the skin in vivo. These results corroborate that while parasite-dependent antibody cytotoxicity is key for neutralizing sporozoites during skin migration, inhibition of liver infection can be efficiently mediated by a non-cytotoxic, binding-related mechanism (Aguirre-Botero et al, 2023). Some basic questions remain unanswered regarding the binding of antibodies to the CSP expressed in the parasites. It is unknown what the possible effect of antibody-induced cross-linking of neighboring CSP molecules in sporozoite neutralization is, an event likely to occur, considering the abundant expression of this protein on the parasite surface. Also, it remains to be determined whether antibody binding to the CSP induces conformational changes that may modify the reactivity of other antibodies or may affect the proteolytic processing of the CSP, which is believed to be necessary for sporozoites to infect hepatocytes (Coppi et al, 2011). New methodological approaches need to be developed to study the effect of antibody binding to the CSP as expressed on the surface of sporozoites, leading to neutralization of parasite infectivity.

The remarkable neutralization capacity of these anti-CSP antibodies indicates that point mutations that could affect the (NPNA)_3_ epitopes of the central repeat domain would not diminish the protective efficacy of specific antibodies. This neutralization activity could only be lost if the entire central repeat domain—more than 100 aa—is modified or replaced. The highly conserved nature of the repeat domain and the remarkable susceptibility of the parasite to neutralizing antibodies specific for these epitopes—which is not observed with antibodies to the N- or C- terminal flanking CSP regions—suggests that antibody binding to the CSP repeat domain disturbs essential, still unknown functions of this parasite surface protein.

## Methods

**Table:** Reagents and tools table

Reagent/resource	Reference or source	Identifier or catalog number
**Experimental models**		
C57Bl/6 J	The Jackson Laboratories	000664
RjOrl:Swiss mice	Elevage Janvier	NA
**Recombinant DNA**		
Synthetic gene NANP4	GenScript	NA
Synthetic gene NANP8		
Synthetic gene NANP12		
pRCPbPfNANP4	Flores-Garcia et al, 2025	NA
pRCPbPfNANP8		
pRCPbPfNANP12		
**Antibodies**		
mAb317	Oyen et al, 2017	NA
mAb311	Oyen et al, 2018	NA
mAb317LALA	Lake Pharma (Curia)	NA
mAb317KA	Lake Pharma (Curia)	NA
Human IgG	SIGMA	
**Oligonucleotides and other sequence-based reagents**		
Pb CSP Fwd	This study	tgttacaatgaaggaaatgataataaattgtat
Pb CSP Rev	This study	aaggtcaaatcttctgctttctt
5’ Int Fwd	This study	tgccctattctcatatttaccac
5’ Int Rev	This study	caccatttttgaaaagattaatttga
**Chemicals, enzymes, and other reagents**		
Nucleofactor 2b Kit	Lonza	VPA-1006
Pyrimethamine	MP Biomedicals	194180
Pierce Fab Preparation kit	ThermoScientific	44985
D-Luciferin Potassium Salt	Revvity (previously PerkinElmer)	122799
HBSS	Gibco	14170-112
PBS	SIGMA	D8537
FBS	Hyclone	SH30071.03
Pen/Strep	Gibco	15140-122
RPMI	Corning	10-041-CV
KpnI	New England Biolabs	
PacI	New England Biolabs	
XhoI	New England Biolabs	
KasI	New England Biolabs	
Matrigel	Corning	
TO-PRO-3	Invitrogen	T3
Glutaraldehyde	Electron Microscopy Sciences, Hatfield, PA	
**Software**		
Living Image 4.7.3	https://www.revvity.com/software-downloads/in-vivo-imaging	
GraphPad Prism 10	https://www.graphpad.com/	
ImageJ	https://imagej.net/ij/	
FlowJo	https://www.flowjo.com/flowjo/overview	
**Other**		
**KI parasite line**	**Sequences from PfCSP**	
PbPf full CS_aa: 25-397_	*…KLKQPADGNPDPNANPNVDPNANPNVDPNANPNVDPNANPNANPNANPNANP…*	
Pf 1× (NPNA)_3 aa:94-116_	…KLKQP*ADGNPDPNANPNANPNANPNANP*PPPPNPNDPPPP…	
Pf 1× (NPNA)_3 aa:198-213_	…KLKQPPPPPNPND…….…NANDPPPPNPND*NANPNANPNANPNANP*PAPPQGNNNPQ…	
Pf 2× (NPNA)_3 aa:94–109 & 214–229_	…KLKQP*NANPNANPNANPNANP*PPPPNPNDPPPPNP………NANDPPPPNPND*NANPNANPNANPNANP*PAPPQGNNNPQ.	
Pf 3× (NPNA)_3 aa:94–141_	…KLKQP*NANPNANPNANPNANPNANPNANPNANPNANPNANPNANPNANPNANP* PPPPNPNDPPPPNPND…	

### Monoclonal antibodies

mAb317 and mAb311 (Oyen et al, 2017) were obtained from PATH (Center for Vaccine Innovation and Access). mAb317 Fc variants were generated at Lake Pharma, Inc (now Curia). Briefly, Human IgG1 Constant Region underwent site mutagenesis, resulting in single amino acid alanine substitution at leucine at positions 234 and 235 (L234A, L235A) and lysine at position 322 (K322A), generating LALA and KA mutants, respectively. Each gene sequence was cloned into LakePharma’s proprietary high-expression mammalian vector, scaled up, and transiently transfected into HEK293 cells. The conditioned media from the transient production run were harvested and clarified by centrifugation and filtration. The supernatant was loaded over a Protein A column pre-equilibrated with binding buffer. Washing buffer was passed through the column until the OD280 value (NanoDrop, Thermo Scientific) was measured to be zero. The target protein was eluted with a low pH buffer, fractions were collected, and the OD280 value of each fraction was recorded. Fractions containing the target protein were pooled and filtered through a 0.2-μm membrane filter. The protein concentration was calculated from the OD280 value and the calculated extinction coefficient.

Control non-specific human IgG was obtained from Sigma Aldrich. The Fab fragments were generated by digestion with papain, following the instructions of the manufacturer (Pierce ^TM^ Fab preparation kit, ThermoFisher). Briefly, 4 mg of full IgGs (human IgG, mAb317 or mAb311) were prepared in digestion buffer, and a total of 0.5 mL of the prepared sample was incubated in a resin containing papain. Incubated for approximately 6 h at 37 °C, maintaining constant mixing. After the incubation, the sample was purified using a NAb Protein A Plus Spin Column to eliminate Fc fragments and undigested IgG After the purification, the Fab samples were dialyzed against PBS in a 10 kDa membrane. Purified mAbs or Fab fragments were diluted to appropriate concentrations in sterile 1× PBS, and concentrations were confirmed using a Nanodrop spectrophotometer. IgG digestion was confirmed by SDS-PAGE.

### Binding of mAbs to mouse and human FcγRI

Binding kinetics of antibody variants to mouse and human FcγRI were determined by biolayer interferometry (BLI) using an Octet system (Sartorius/ForteBio). Recombinant biotinylated mouse FcγRI was captured on streptavidin biosensor tips, whereas recombinant His-tagged human FcγRI was immobilized on HIS1K biosensor tips. All sensors were equilibrated in kinetics buffer (PBS supplemented with 0.02% Tween-20 and 0.1% BSA) prior to analysis. After baseline establishment, FcγRI was loaded to a target response of approximately 0.8–1.2 nm, followed by a stabilization step in kinetics buffer. Association was monitored by dipping sensors into wells containing serial dilutions of each antibody variant (1.37–1000 nM) for 300 s, and dissociation was measured for 300 s in kinetics buffer. Reference sensors lacking ligand and analyte were included for double-reference subtraction. Sensorgrams were processed and aligned using Octet data analysis software. Kinetic parameters were obtained by global fitting to a 1:1 Langmuir binding model to determine the association rate constant (k_on), dissociation rate constant (k_off), and equilibrium dissociation constant (K_D). All experiments were performed at 25 °C with agitation at 1000 rpm.

### Mice

Six to seven-week-old female C57BL/6 mice (Jackson Laboratories) were maintained at the animal facility of the Bloomberg School of Public Health, Johns Hopkins University. Every procedure performed was approved by the Animal Care and Use Committee (Protocol MO21H417). For experiments displayed in Figs. 3 and 6, female *Anopheles* mosquitoes were fed on infected 20 g RjOrl:Swiss mice (Elevage Janvier). Mice were kept in the animal facility of the CEPIA at the Institut Pasteur in Paris, accredited by the French Ministry of Agriculture for performing experiments on rodents. These experiments were performed according to French and European regulations and were approved by the Ethics Committee #89 (references MESR 01324, APAFIS#32422-2021071317049057 v2, APAFIS #32989-2021091516594748 v1).

### Generation of transgenic *P. berghei* parasite lines expressing the (NPNA)_3_ epitopes

Pb parasites (strain ANKA, 676m1c11, MRA-868) expressing green fluorescent protein (GFP) and luciferase were obtained from MR4, ATCC. Pyrimethamine plasmids (pR-CSPFL) encoding the different versions of PbCSP, along with the PbCSP signal sequence, (Reagents Table) were obtained after ligations assay of synthetic genes generated at GenScript. Plasmid transfections were performed as previously described (Janse et al, 2006) to generate the different transgenic parasite lines. All plasmids include the hDHFR selection cassette and *csp* 5’ and 3’ UTRs. pR-CSPFL was excised with XhoI and KasI and transfected into schizont cultures of Pb parasites by electroporation using an Amaxa Nucleofactor (Espinosa et al, 2017). Transfected parasites were injected i.v. into Swiss Webster mice and selected with pyrimethamine in drinking water (7 mg/mL). The pyrimethamine-resistant parasites were cloned by limiting dilution in mice. All isolated clones were sequenced after PCR of the transgene using primers that amplified the entire 800-900 bp of the inserted modified CSP gene, (PbCS Fwd; tgttacaatgaaggaaatgataataaattgtat, PbCS Rev; aaggtcaaatcttctgctttctt) (Fig EV2A).

### Parasites

Sporozoites from Pb expressing GFP-luciferase, and the different CSP versions were obtained from salivary glands of infected mosquitoes, as previously described (Flores-Garcia et al, 2019). Briefly, *Anopheles stephensi* mosquitoes were fed on parasite-infected mice after confirming the presence of gametocyte exflagellation (Espinosa et al, 2013). After infection, mosquitoes were maintained in an incubator at 19–20 °C and supplied with a sterile cotton pad soaked in 10% sucrose, changed every 48 h. Sporozoites were harvested 19–22 days after blood feeding.

### mAb-mediated inhibition of SPZ motility in vitro

The in vitro motility assays were performed as previously described (Flores-Garcia et al, 2021). Briefly, freshly dissected sporozoites were suspended HBSS- 2% FBS. The parasite adjusted to 10,000 Spz/μL and 20 μL were allowed to settle on a 35 mm glass-bottom Mattek dish for 10 min on ice. Motility was subsequently assayed using time-lapse microscopy at 37 °C, with recordings made using an Olympus IX-71 inverted wide-field fluorescence microscope with a PCO complementary metal-oxide-semiconductor (CMOS) camera driven by DeltaVision software (Applied Precision, Seattle, WA). Sporozoites were imaged using an LED light source and FITC filter. A field of view containing approximately 30 sporozoites was chosen, and videos were taken using a 5 s time-lapse for 15 min total. mAb317 and 317 Fab were dialyzed and re-suspended in HBSS with 2% FBS, added directly to the dish containing the sporozoite suspension. Parasites continuously moving in a circle before mAb addition were identified as motile, and as non-motile only if their circular movement came to a complete stop 15 min after mAb exposure. Motility was assessed with the assistance of ImageJ software. The percentage of non-motile parasites was calculated as follows: non-motile sporozoites/motile sporozoites ×100.

To determine sporozoite speed, average velocity was assessed as previously described (Aguirre-Botero et al, 2023). In total, 5000 sporozoites were re-suspended in PBS containing 5% FCS and 10 µg/mL of mAb317 or the indicated concentration of 317 Fab. The suspension was transferred to an 18-well slide (iBidi) and centrifuged at 400× *g* for 3 min at 4 °C. The slide was incubated at 37 °C, 5% CO_2_ for 3 min in the incubation chamber (Incubation System S, Zeiss) of an inverted epifluorescence wide-field microscope (Axio Observer, Zeiss). Time-lapse movies were then recorded for 2–4 min at a rate of one image per second with an EC “Plan- Neofluar” 10×/0.3 objective (Zeiss) using a 470 nm LED and a matching filter cube (43HE, Zeiss) to excite and detect GFP and thus visualize sporozoites. The average sporozoite velocity was calculated from the circular gliding over the first 2 min of the acquisition using the MTrack2 plug-in from Fiji.

### In vitro sporozoite cytotoxicity assay in suspension and Matrigel

Antibody lethality in suspension and in Matrigel was measured as previously described (Aguirre-Botero et al, 2023). For the assay in suspension, 10,000–12,000 sporozoites were incubated at 37 °C for 45 min with different concentrations of 317 IgG/Fab in the presence of 10% FCS. Samples were next transferred on ice and incubated for 10 min with 1 µM TO-PRO-3 (T3, Invitrogen). Samples were then diluted ten times with cold PBS before the acquisition on a CytoFLEX S flow cytometer (Beckman Coulter). At least 5000 events in the sporozoite gate were acquired. Viability was defined as the percentage of GFP + PI- sporozoites to the sum of GFP + T3-, GFP- T3+, and GFP + T3+ sporozoites. The CSPR was analyzed by the forward scatter-width signal intensity (FSC-W). CSPR span was determined by subtracting the bottom from the top value of the sigmoidal FSC-W curve. Data were analyzed using the CytExpert 2.0 software (Beckman Coulter). For the 3D assay, 10,000–12,000 sporozoites in PBS were mixed on ice with Corning Matrigel matrix (Corning) in a 1:4 ratio (80% Matrigel). Following polymerization at 37 °C for 5 min, one volume of mAb317 or 317 Fab in 20% FCS/PBS was added (10% FCS final concentration). After 45 min at 37 °C, the samples were placed on ice for 5 min. Then, T3 was added for 10 min on ice and diluted 11 times with cold PBS. The samples were then analyzed by flow cytometry. The number of PbPf parasites in the control was used to normalize the sporozoite recovery across samples. Viability was defined as the percentage of PbPf GFP+ sporozoites compared to the control. The IgG and Fab concentrations were matched to have the same number of molecules. mAb317 concentrations: 100, 30, 10, 3 µg/mL. 317 Fab concentrations: 67, 20, 6.7, 2 µg/mL. All data were analyzed using the CytExpert 2.0 software (Beckman Coulter).

### Sporozoite immunofluorescence assay

Sporozoites were treated with 100 µg/mL of IgG or 67 µg/ml of Fab and 10% FCS/PBS. After 45 min of incubation at 37 °C, the samples were placed on ice, transferred to an 18-well iBidi slide, and fixed with 2% Formaldehyde (Thermo Fisher Scientific) for 20 min at room temperature. The samples were then blocked with 1% BSA in PBS and incubated with 4 mg/mL Alexa Fluor™ 350-conjugated goat anti-human IgG (H + L) antibody (Invitrogen) in PBS containing 1% BSA and Spy-tubulin in 1% BSA/PBS for 1 h at room temperature. After washing, the wells were filled with mounting medium (iBidi), and images were taken using a Zeiss Axio Observer inverted microscope and analyzed with Fiji software.

### Electron microscopy

For thin-section transmission EM, 1.1 million sporozoites were re-suspended in 210 µL HBSS with 2% serum. 50 µL of sporozoite suspension was added to four separate tubes. One tube received antibody (e.g., mAb317), the second tube received 317 Fab, and the third tube received control mAb1245. All antibodies were used at 75 µg/mL final concentration. After antibody addition, parasites were incubated at 37 °C for 10 min before fixation in 2.5% glutaraldehyde (Electron Microscopy Sciences, Hatfield, PA) in 0.1 M sodium cacodylate buffer (pH 7.4) for 1 h at room temperature. Fixed parasites were then processed as previously described (Coppens and Joiner, 2003) and examined with a Philips CM120 electron microscope (Eindhoven, the Netherlands) under 80 kV.

### Sporozoite i.v. challenge

Estimation of parasite liver burden by bioluminescence was performed as previously described (Flores-Garcia et al, 2019). Briefly, transgenic SPZ freshly harvested from mosquito salivary glands were suspended in 2% FCS-HBSS media and adjusted to 10,000 SPZ/ml. Unless otherwise noted, mice were challenged with 2000 SPZ i.v. in the tail vein, 16 h after passive immunization of mAbs by i.v. injection into the tail vein. 42 h after SPZ challenge, mice were injected intraperitoneally with 100 µL of D-Luciferin (30 mg/mL) and anesthetized in an isoflurane chamber. Once mice were immobilized, bioluminescence in their livers was measured for 5 min using an IVIS Spectrum in vivo imaging system (PerkinElmer).

### Mosquito bite challenge

Mosquito bite challenge in mice was performed as previously described (Flores-Garcia et al, 2019). Briefly, 6 h after passive transfer of mAbs into the tail vein i.v., mice were exposed to the bites of five infected mosquitoes obtained from a population of mosquitoes that was 80% infected. Mice were anesthetized with 2% Avertin and placed on top of cages containing the infected mosquitoes for 10 min. Starting 4 days after the mosquito bite challenge, Giemsa-stained blood smears were examined daily by light microscopy to look for blood-stage parasites until 12 days post-challenge.

## Supplementary information

Peer Review File

Source data Fig. 1

Source data Fig. 2

Source data Fig. 3

Source data Fig. 4

Source data Fig. 5

Source data Fig. 6

Expanded View Figures

## Data Availability

This study includes no data deposited in external repositories. The source data of this paper are collected in the following database record: biostudies:S-SCDT-10_1038-S44319-026-00788-3.
