# Supplementary figures and images for: Relevance of the antibody Fc fragment and epitope valency in protection against malaria sporozoites
Source: EMBO Rep. 2026 Apr 27;27(12):3340–58. doi: 10.1038/s44319-026-00788-3 (PMC13303866; doi:10.1038/s44319-026-00788-3)

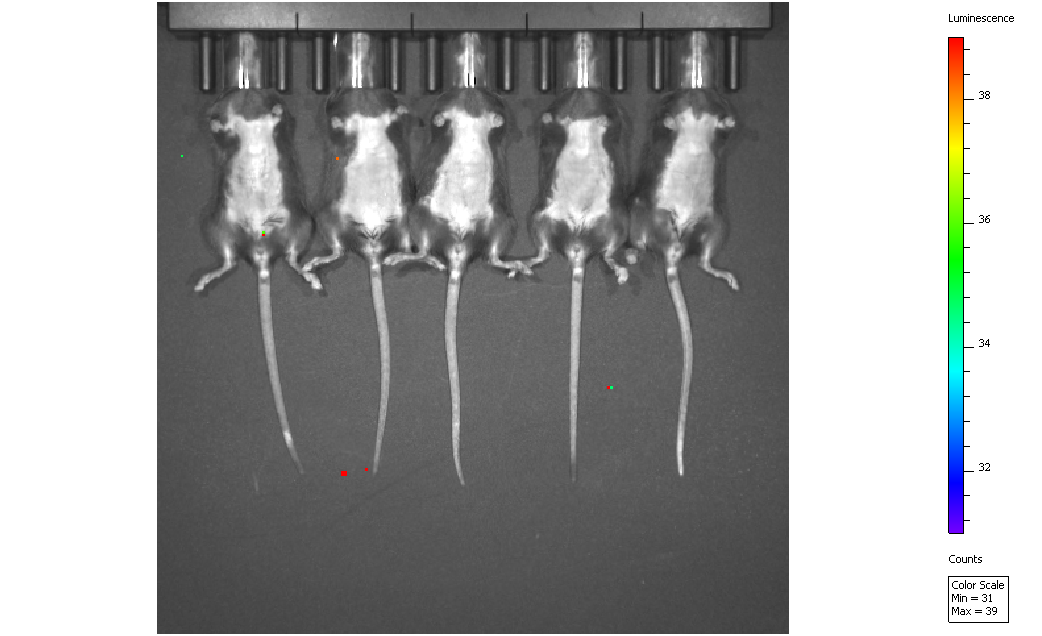

Supplement: Supplementary file 2 — Source data Fig. 1 [file 44319_2026_788_MOESM2_ESM.zip › IVIS Images Figure 1D/AB317 666 nmolar.tif]

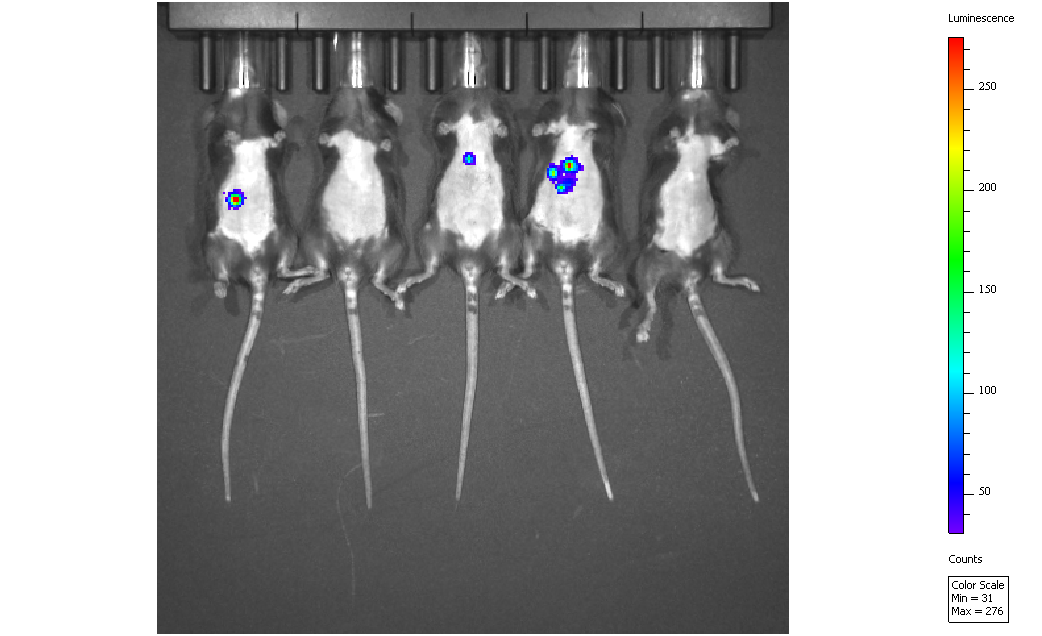

Supplement: Supplementary file 2 — Source data Fig. 1 [file 44319_2026_788_MOESM2_ESM.zip › IVIS Images Figure 1D/317Fab 222 nmolar.tif]

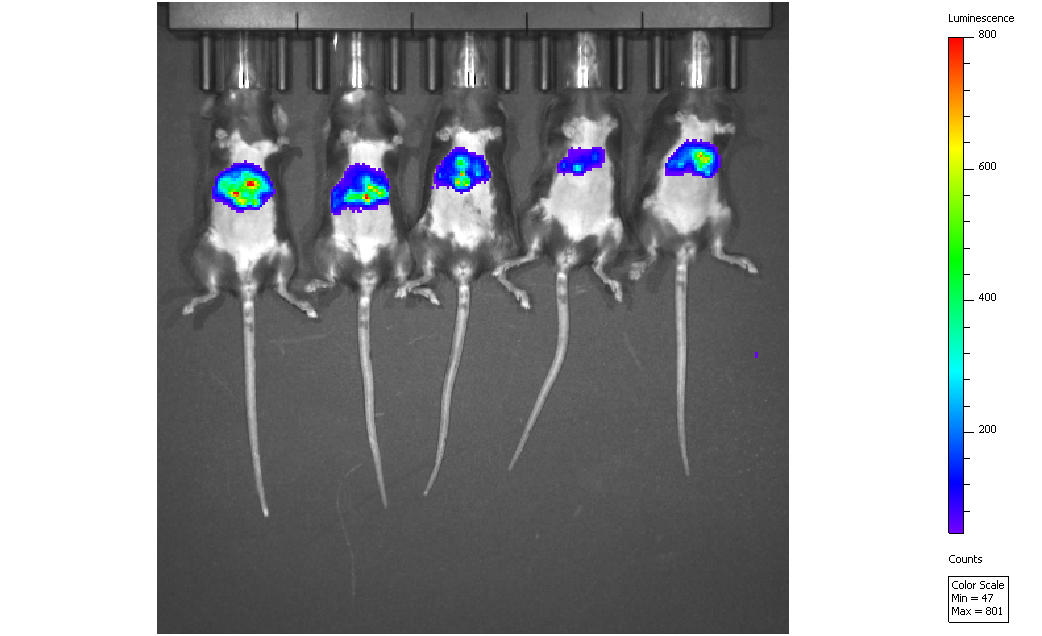

Supplement: Supplementary file 2 — Source data Fig. 1 [file 44319_2026_788_MOESM2_ESM.zip › IVIS Images Figure 1D/317Fab 74 nmolar.tif]

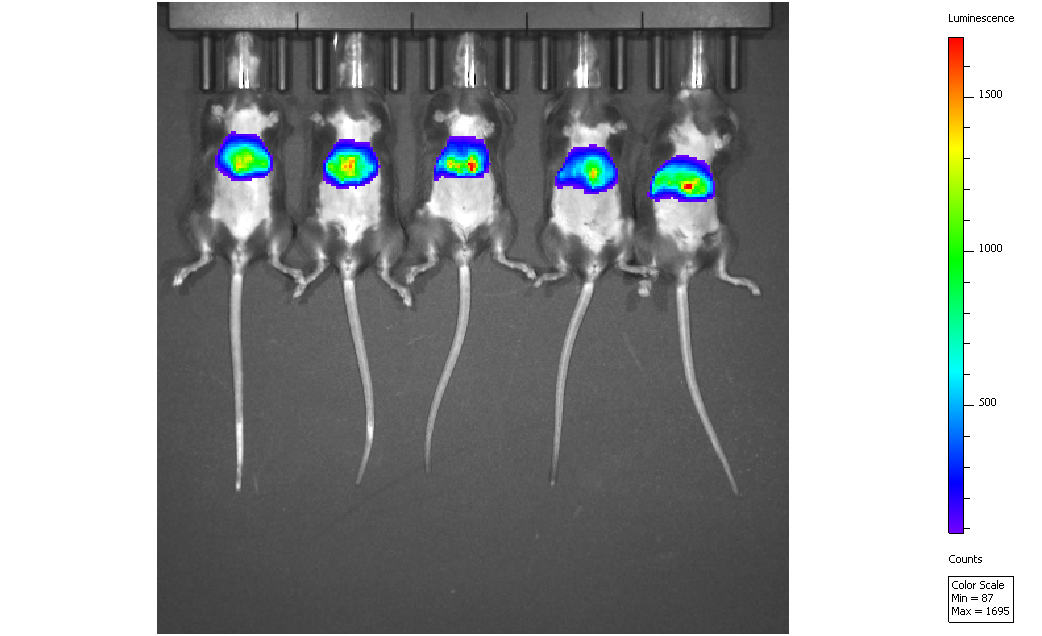

Supplement: Supplementary file 2 — Source data Fig. 1 [file 44319_2026_788_MOESM2_ESM.zip › IVIS Images Figure 1D/Naive.tif]

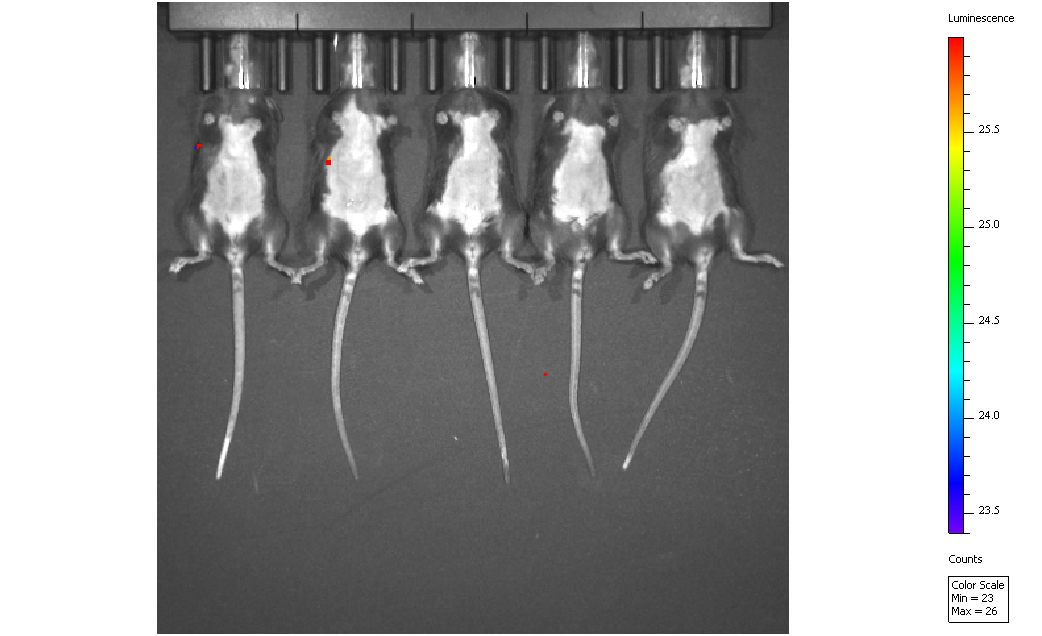

Supplement: Supplementary file 2 — Source data Fig. 1 [file 44319_2026_788_MOESM2_ESM.zip › IVIS Images Figure 1D/317Fab 666 nmolar.tif]

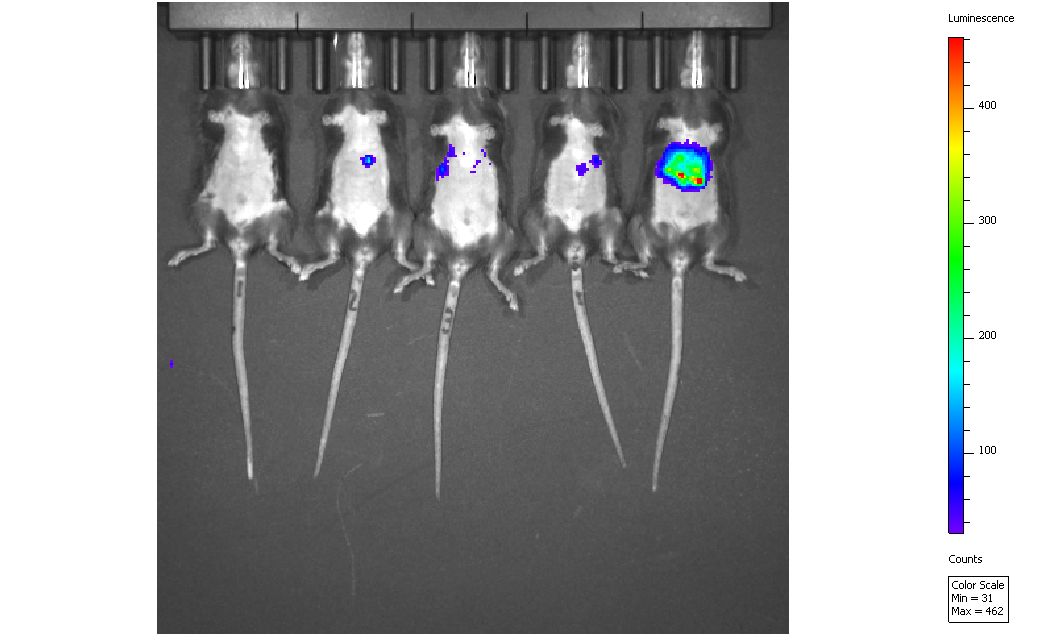

Supplement: Supplementary file 2 — Source data Fig. 1 [file 44319_2026_788_MOESM2_ESM.zip › IVIS Images Figure 1D/AB317 74 nmolar.tif]

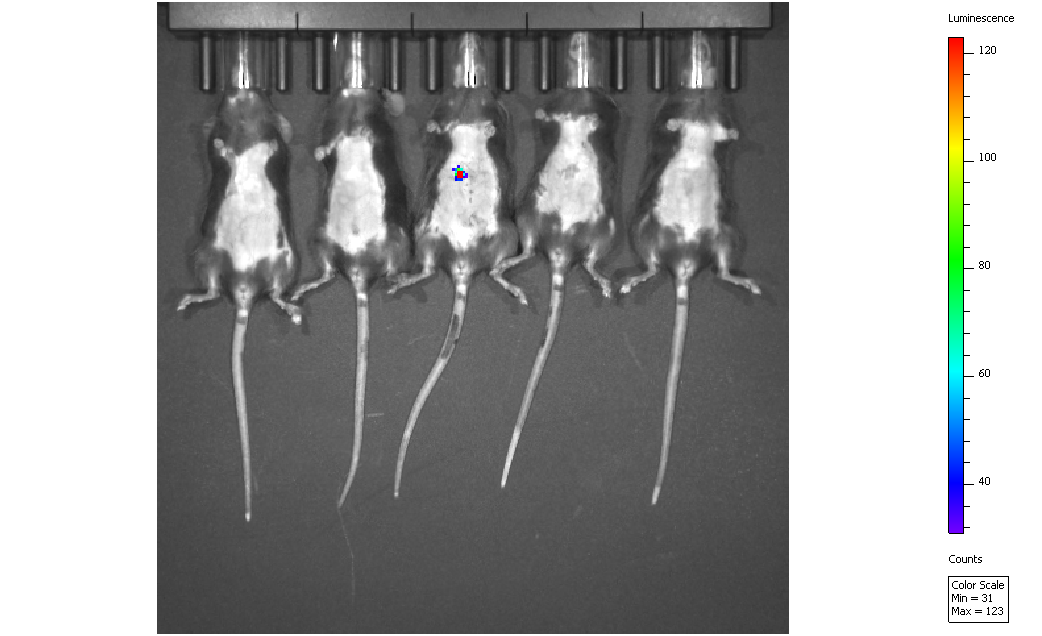

Supplement: Supplementary file 2 — Source data Fig. 1 [file 44319_2026_788_MOESM2_ESM.zip › IVIS Images Figure 1D/AB317 222 nmolar.tif]

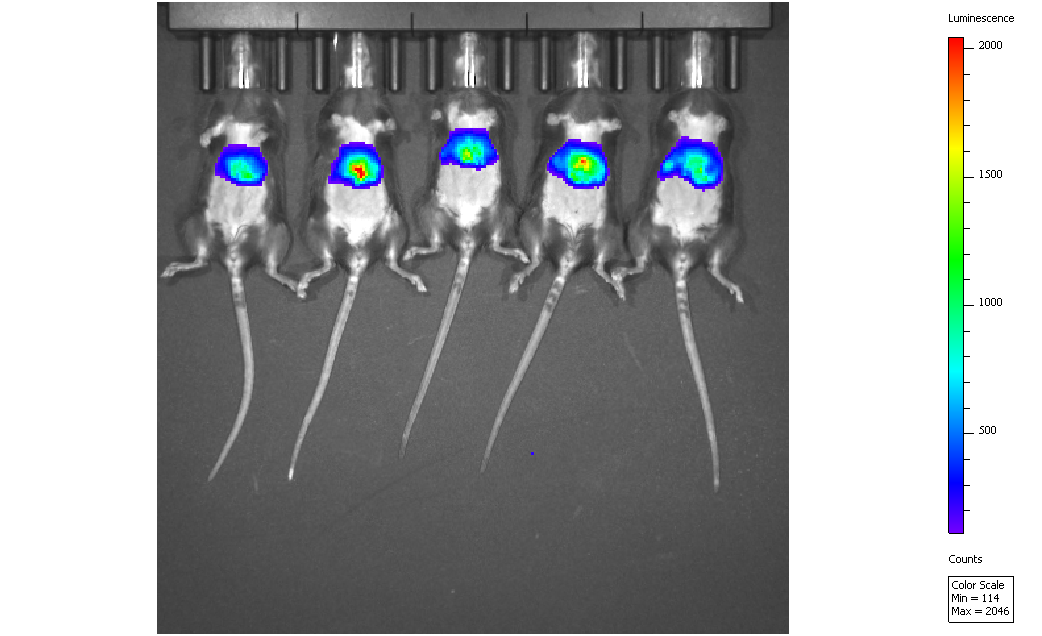

Supplement: Supplementary file 2 — Source data Fig. 1 [file 44319_2026_788_MOESM2_ESM.zip › IVIS Images Figure 1D/Human Fab 666 nmolar.tif]

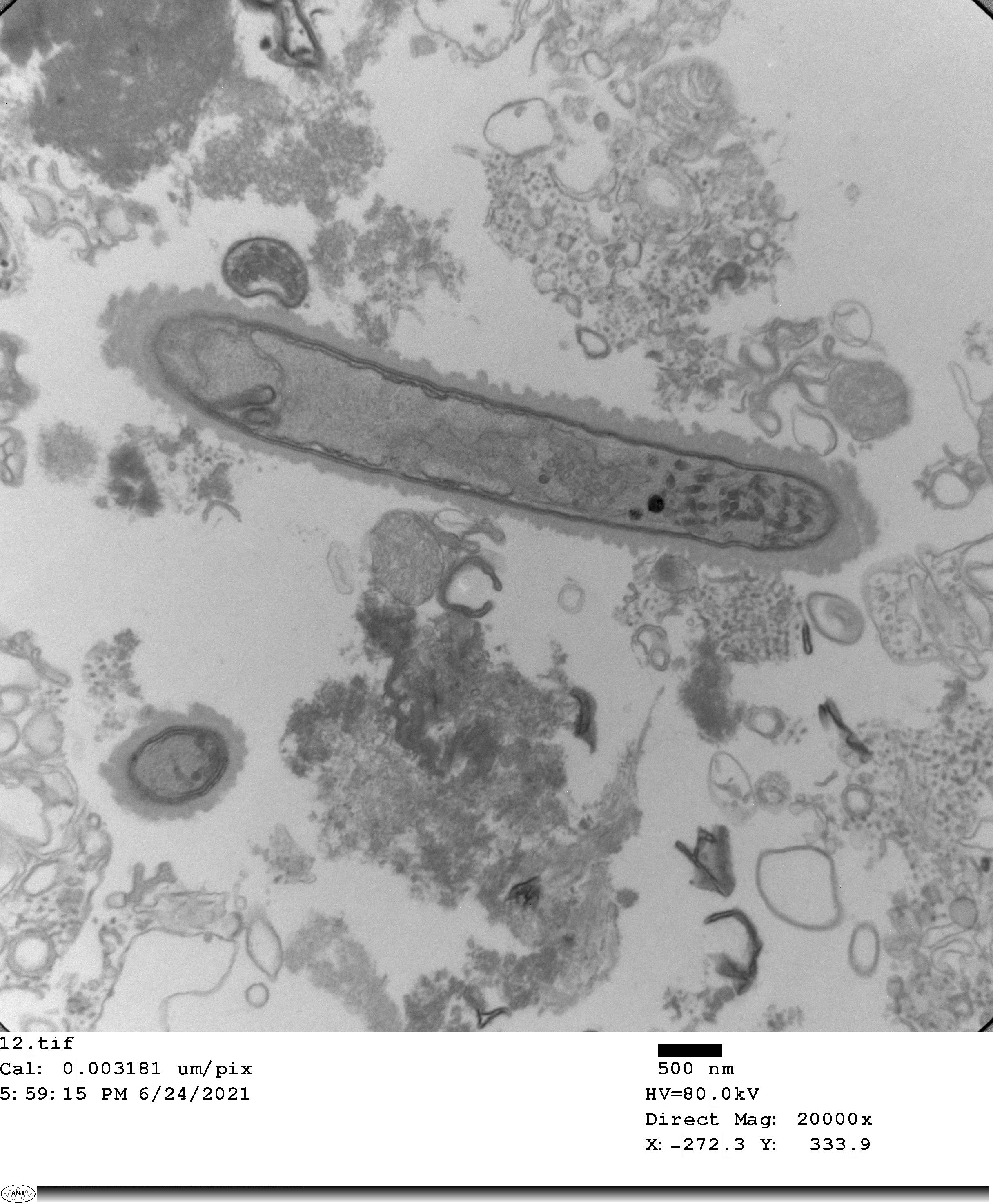

Supplement: Supplementary file 3 — Source data Fig. 2 [file 44319_2026_788_MOESM3_ESM.zip › Figure 2/2Bb.tiff]

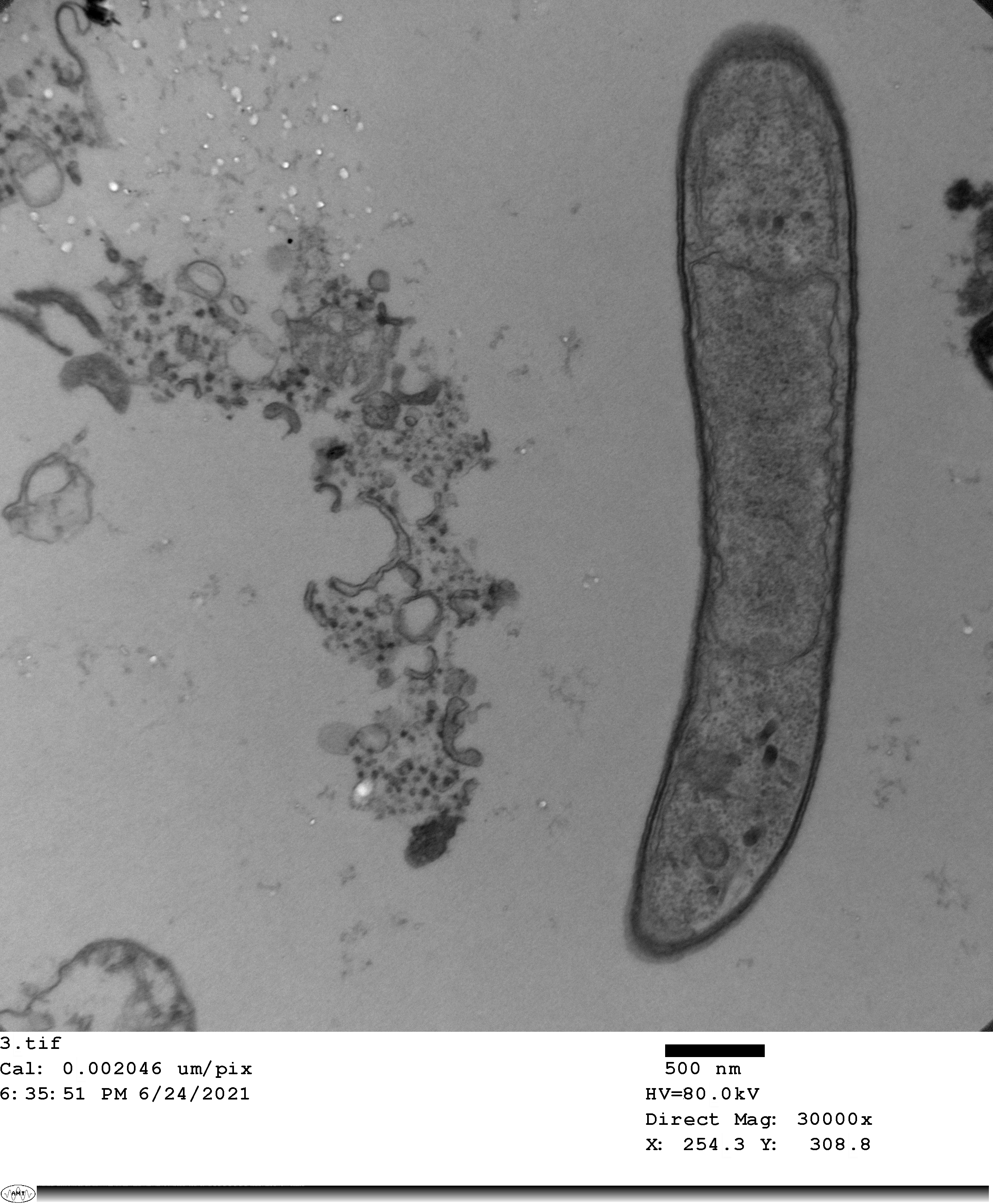

Supplement: Supplementary file 3 — Source data Fig. 2 [file 44319_2026_788_MOESM3_ESM.zip › Figure 2/2Ab.tiff]

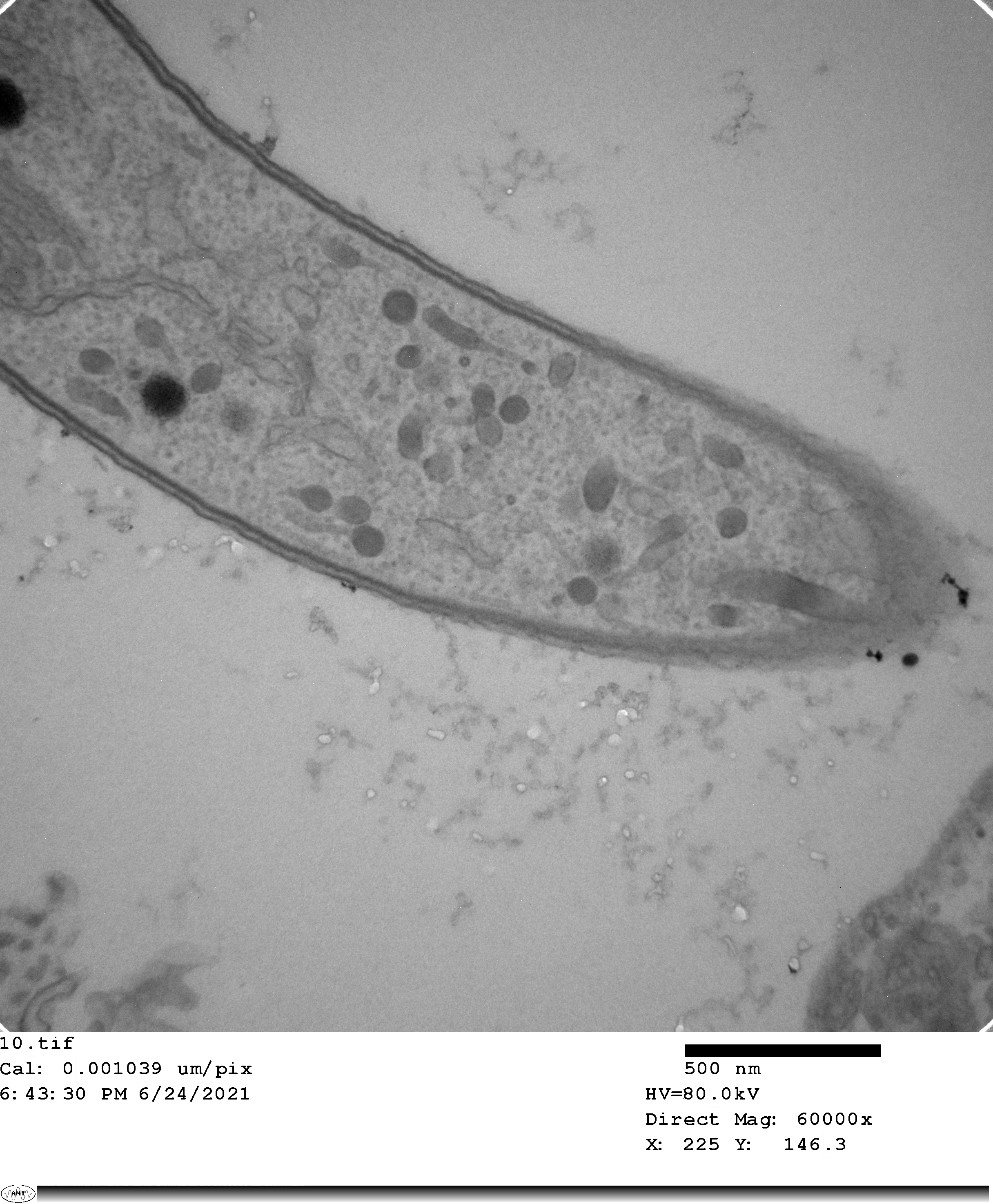

Supplement: Supplementary file 3 — Source data Fig. 2 [file 44319_2026_788_MOESM3_ESM.zip › Figure 2/2Cb.tiff]

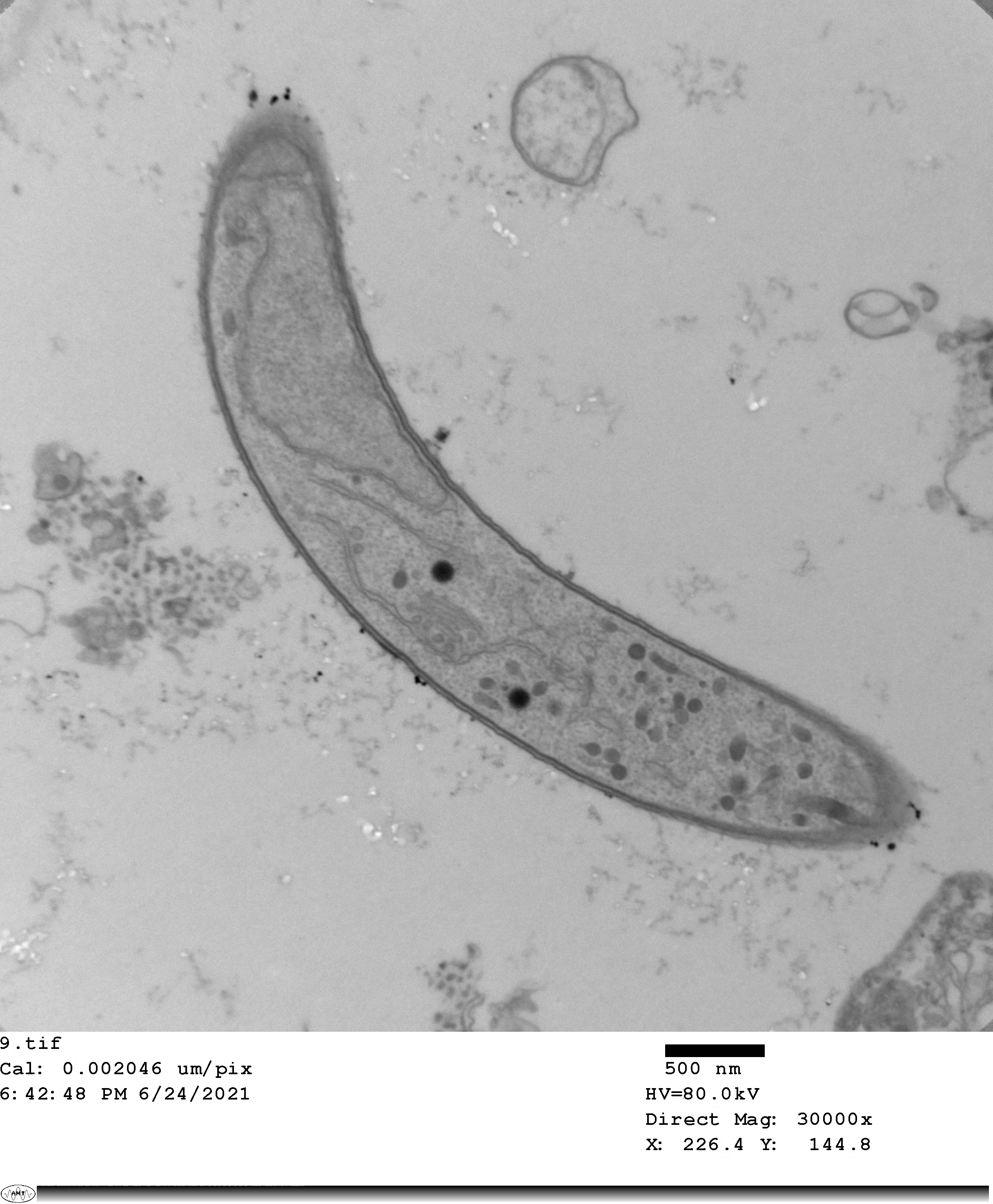

Supplement: Supplementary file 3 — Source data Fig. 2 [file 44319_2026_788_MOESM3_ESM.zip › Figure 2/2Ca.tiff]

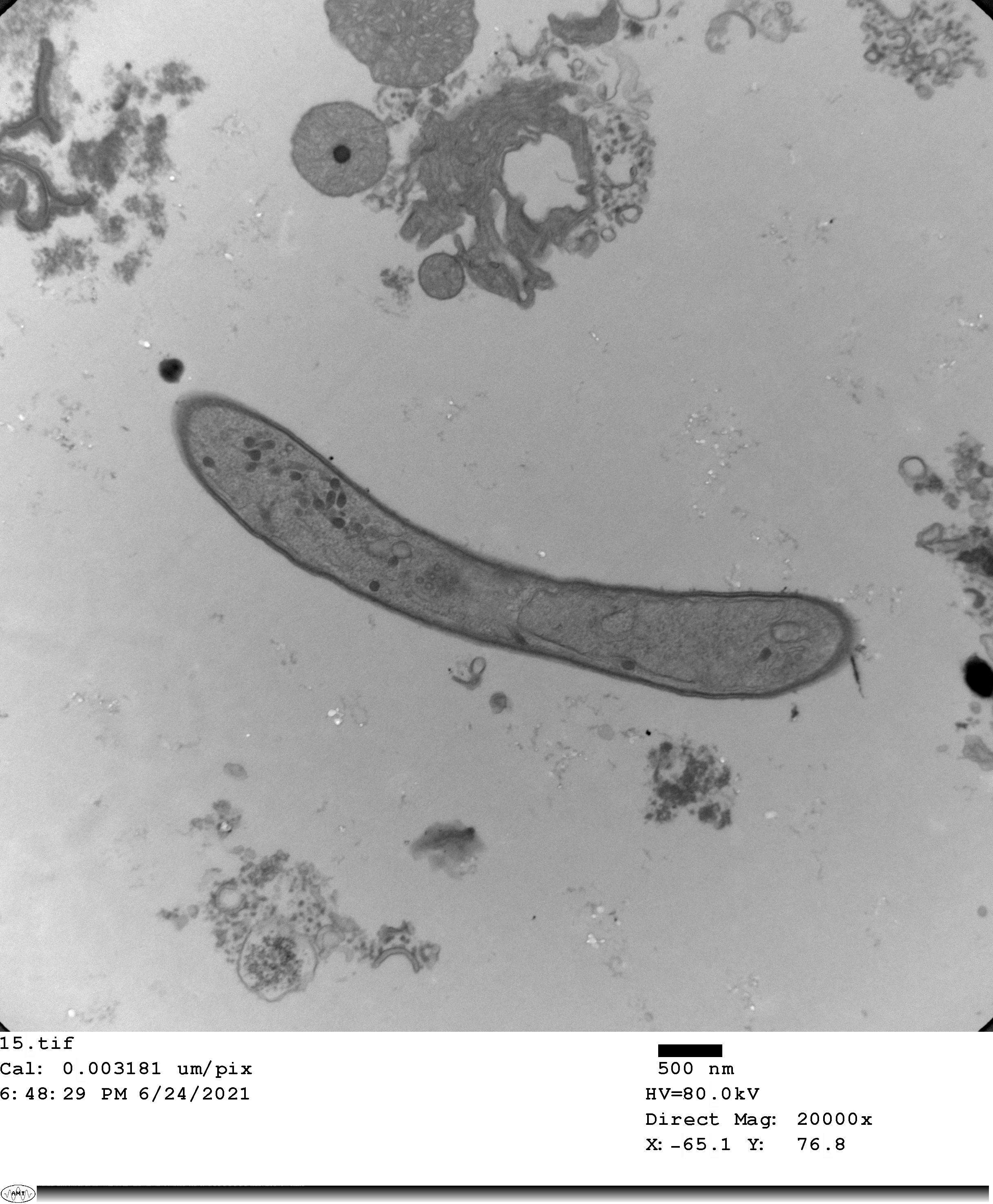

Supplement: Supplementary file 3 — Source data Fig. 2 [file 44319_2026_788_MOESM3_ESM.zip › Figure 2/2Aa.tiff]

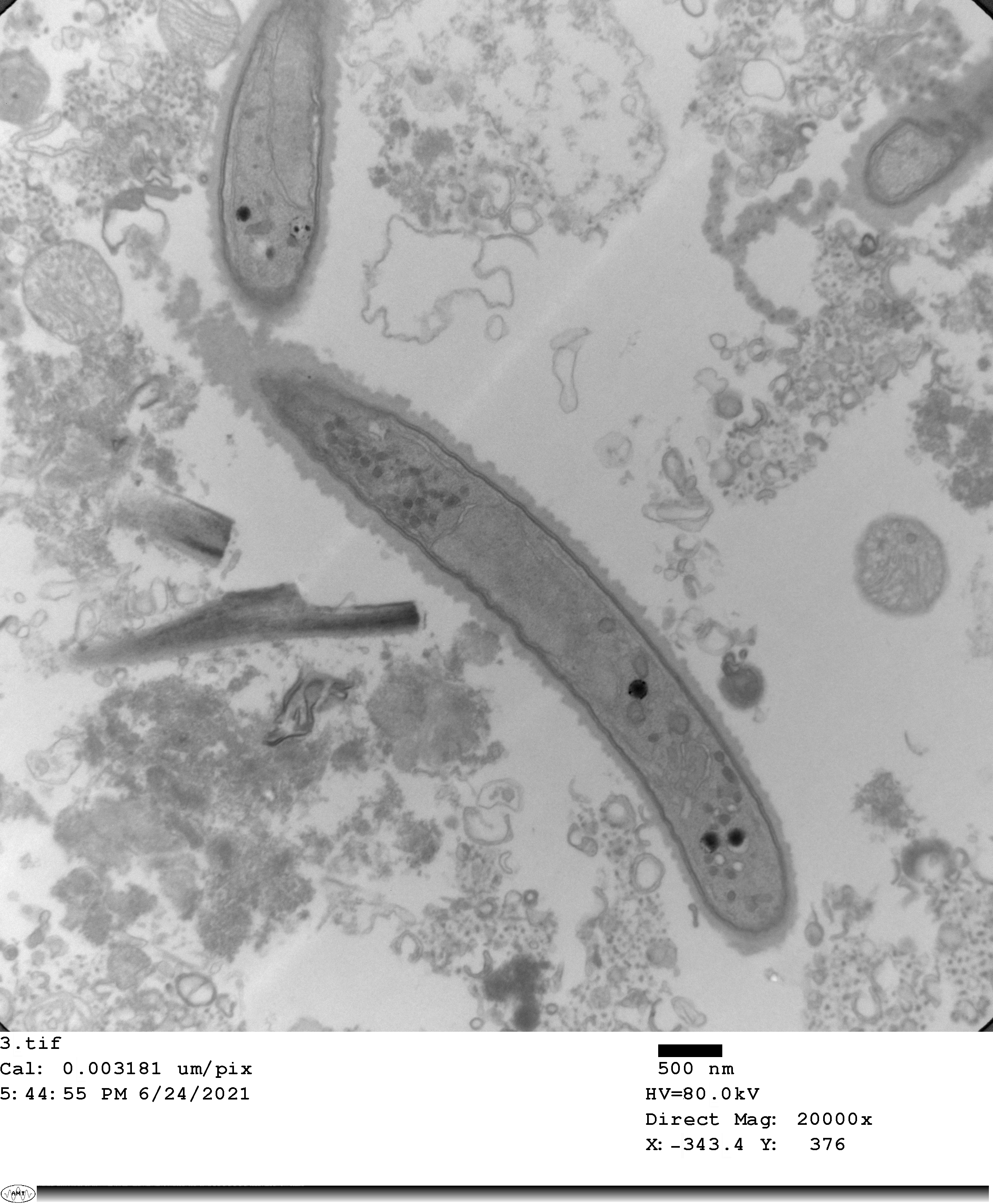

Supplement: Supplementary file 3 — Source data Fig. 2 [file 44319_2026_788_MOESM3_ESM.zip › Figure 2/2Ba.tiff]

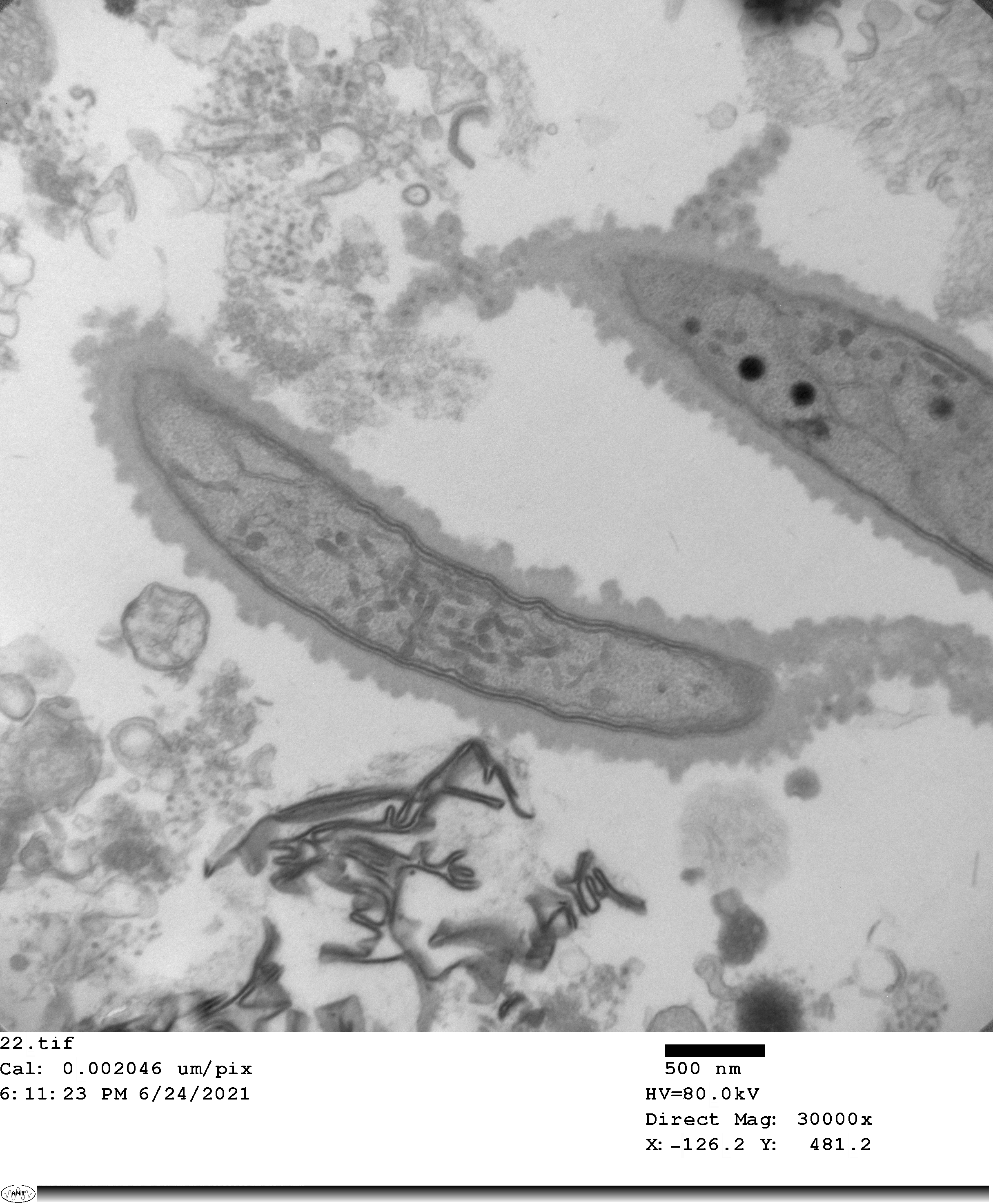

Supplement: Supplementary file 3 — Source data Fig. 2 [file 44319_2026_788_MOESM3_ESM.zip › Figure 2/2Bc Bd.tiff]
