# Supplementary material for: Relevance of the antibody Fc fragment and epitope valency in protection against malaria sporozoites
Source: EMBO Rep. 2026 Apr 27;27(12):3340–58. doi: 10.1038/s44319-026-00788-3 (PMC13303866; doi:10.1038/s44319-026-00788-3)
Supplement: Supplementary file 8 — Expanded View Figures [file 44319_2026_788_MOESM8_ESM.pdf]

Expanded View Figures

A

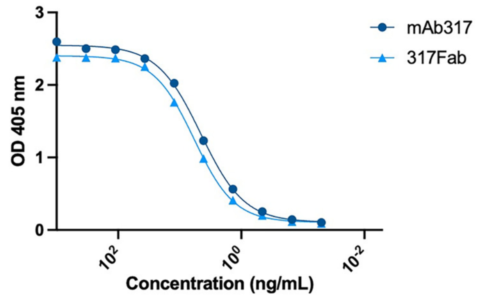

B

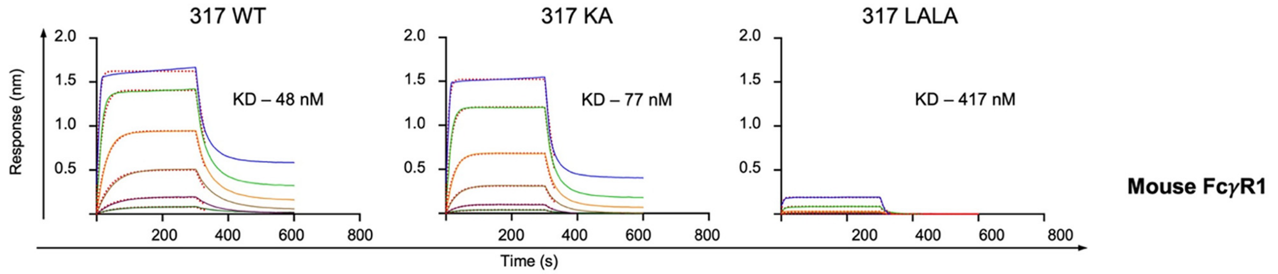

Antibody	KD (M)	KD Error	ka (1/Ms)	ka Error	kdis (1/s)	kdis Error
317 WT	4.79E-08	6.22E-10	4.10E+05	4.21E+03	1.96E-02	1.56E-04
317 KA	7.72E-08	9.67E-10	3.89E+05	4.21E+03	3.00E-02	1.89E-04
317 LALA	4.17E-07	1.78E-08	1.85E+05	7.38E+03	7.69E-02	1.18E-03

The error represents standard error of the fitted parameter.

C

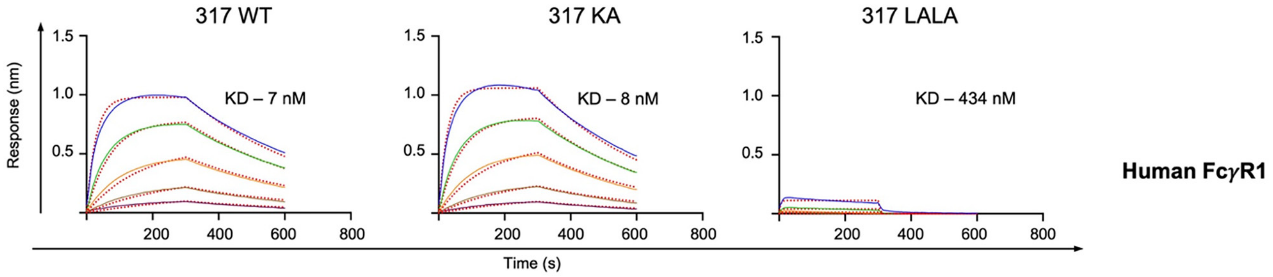

Antibody	KD (M)	KD Error	ka (1/Ms)	ka Error	kdis (1/s)	kdis Error
317 WT	7.64E-09	7.90E-11	3.13E+05	2.80E+03	2.39E-03	1.24E-05
317 KA	8.68E-09	7.92E-11	3.29E+05	2.67E+03	2.85E-03	1.18E-05
317 LALA	4.34E-07	2.60E-07	4.34E+05	2.57E+05	1.88E-01	1.66E-02

The error represents standard error of the fitted parameter.

Figure EV1. ELISA of mAb317 and 317 Fab binding to CSP and Biolayer interferometry of antibody binding to FcγRI.

(**A**) mAb317 (dark blue circles) and 317 Fab (light blue triangles) show the same binding activity to rCSP. ELISA assay using rCSP as antigen was used to evaluate the binding activity of mAb317 and 317 Fab fragments, results of a test run in duplicates. (**B**, **C**) Biolayer interferometry (BLI) analysis of antibody variant binding to mouse and human FcγRI. Binding to mouse (**B**) or human (**C**) FcγRI was assessed by biolayer interferometry (BLI) at physiological pH (7.4). Representative sensorgrams are shown with experimental data overlaid with the global fit (red dotted line) from one experiment run in duplicate. Equilibrium dissociation constants (KD, nM) are indicated on each panel.

© The Author(s)

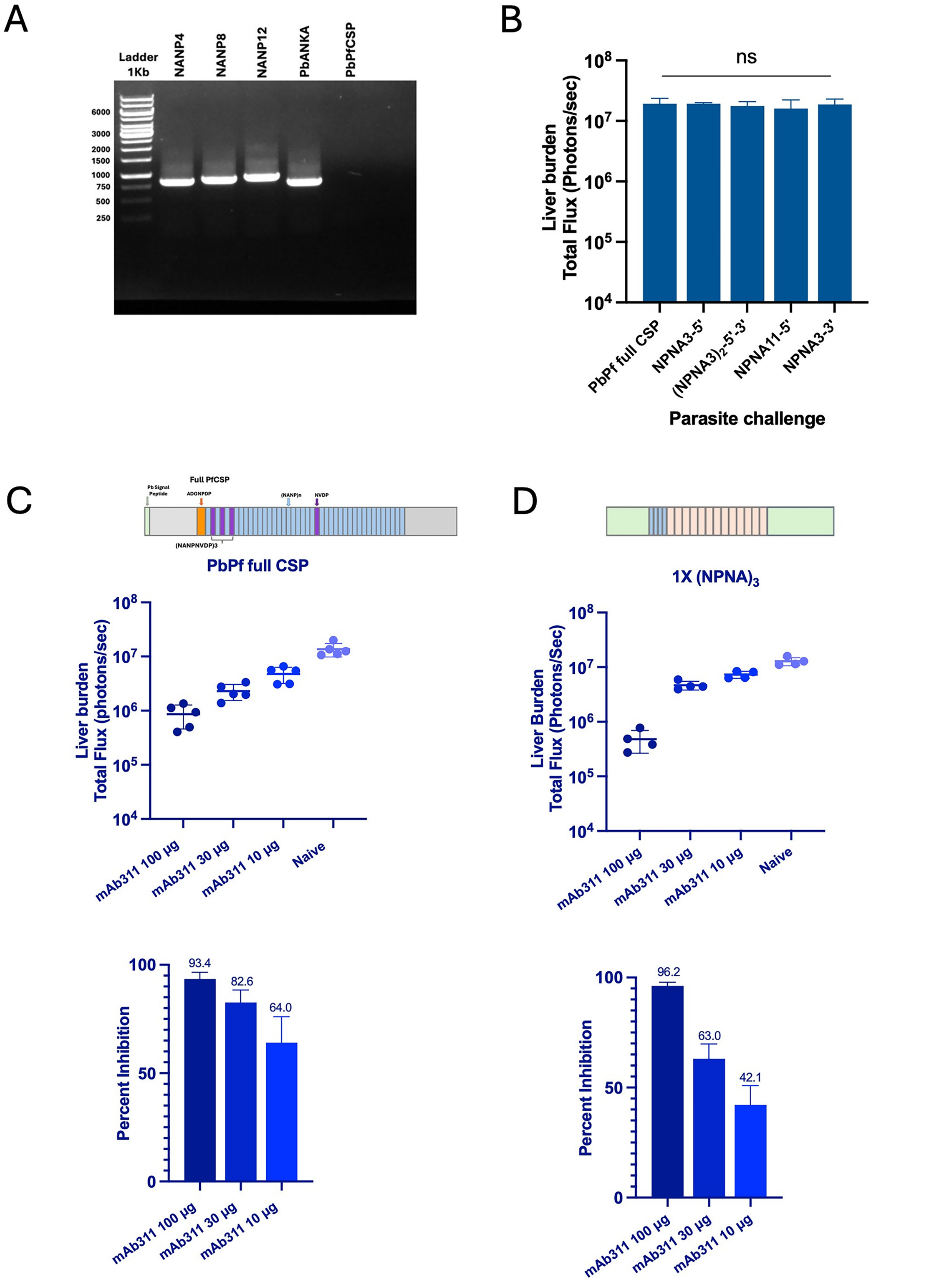
A
Ladder 1Kb
NANP4
NANP8
NANP12
PbANKA
PbPfCSP
6000
3000
2000
1500
1000
750
500
250
B
ns
Liver burden
Total Flux (Photons/sec)
10^8
10^7
10^6
10^5
10^4
PbPf full CSP
NPNA3-5'
(NPNA3)2-5'-3'
NPNA11-5'
NPNA3-3'
Parasite challenge
C
Full PfCSP
Pb Signal Peptide
ADGNPDP
(NANP)n
NVDP
(NANPNVDP)3
PbPf full CSP
Liver burden
Total Flux (photons/sec)
mAb311 100 µg
mAb311 30 µg
mAb311 10 µg
Naive
Percent Inhibition
93.4
82.6
64.0
100
50
0
D
1X (NPNA)3
Liver Burden
Total Flux (Photons/Sec)
96.2
63.0
42.1

Figure EV2. Characterization of the transgenic parasite lines regarding the gene expression and infectivity.

(**A**) The new transgenic sporozoite lines show a size shift in the modified CSP gene and (**B**) have comparable infectivity while expressing different numbers of NANP repeats. (**C**) Anti-CSP mAb311 confers protection when the parasites express 1 or 11 $(NPNA)_3$ epitope (**A**) PCR products showing different sizes of the modified CSP gene from DNA isolated from the new transgenic parasites. (**B**) Female C57BL/6 mice (5 mice/group) were injected i.v. with 2000 transgenic sporozoites. The graph shows parasite bioluminescence in the liver expressed by the transgenic parasites, mean with standard deviation. Two-way ANOVA showed no statistical differences in infectivity among parasite lines. C57BL/6 mice ($n = 4$–5) were injected i.v. with 100, 30, or 10 µg of mAb311 and i.v. challenged 6 h later with 2000 transgenic sporozoites expressing (**C**) PbPf full CSP, (**D**) $1\times(NPNA)_3$. Upper panels: parasite burden in the liver (total flux, photons/s); bottom panels: percent inhibition in liver burden mediated by mAb311. The scatterplots show the mean and standard deviation of one of two biological replicates.

© The Author(s)
