# Supplementary material for: Relevance of the antibody Fc fragment and epitope valency in protection against malaria sporozoites
Source: EMBO Rep. 2026 Apr 27;27(12):3340–58. doi: 10.1038/s44319-026-00788-3 (PMC13303866; doi:10.1038/s44319-026-00788-3)
Supplement: Supplementary file 1 — Peer Review File [file 44319_2026_788_MOESM1_ESM.pdf]

EMBO reports

Relevance of the antibody Fc fragment and epitope valency in protection against malaria sporozoites

Fidel Zavala, Yevel Flores-Garcia, Manuela Aguirre-Botero, Emily Locke, Minah Park, Shamika Mathis-Torres, Berenice Salgado-Jimenez, Beejan Asady, Isabelle Coppens, Rogerio Amino, Sonia Herrera-Arevalo, Prabhanshu Tripathi, and Anisha Gladston

Corresponding author(s): Fidel Zavala (fzavala1@jhu.edu) , Rogerio Amino (roti@pasteur.fr)

Review Timeline:

Submission Date:	10th Oct 25
Editorial Decision:	19th Nov 25
Revision Received:	27th Feb 26
Editorial Decision:	27th Mar 26
Revision Received:	9th Apr 26
Accepted:	16th Apr 26

Editor: Achim Breiling

Transaction Report:

(Note: With the exception of the correction of typographical or spelling errors that could be a source of ambiguity, letters and reports are not edited. Depending on transfer agreements, referee reports obtained elsewhere may or may not be included in this compilation. Referee reports are anonymous unless the Referee chooses to sign their reports.)

Dear Dr. Zavala,

Thank you for the transfer of your manuscript to EMBO reports. I have now received reports from the three referees that were asked to evaluate your study, which can be found at the end of this email. As you will see, the referees think that these findings are of high interest. However, they have several comments, concerns, and suggestions, indicating that a major revision of the manuscript is necessary to allow publication of the study in EMBO reports. As the reports are below, and all the referee concerns need to be addressed, I will not detail them here.

Given the constructive referee comments, I would thus like to invite you to revise your manuscript with the understanding that the concerns of the referees must be addressed in the revised manuscript and/or in a detailed point-by-point response. Acceptance of your manuscript will depend on a positive outcome of a second round of review. It is EMBO reports policy to allow a single round of revision only and acceptance of the manuscript will therefore depend on the completeness of your responses included in the next, final version of the manuscript.

Revised manuscripts should be submitted within three months of a request for revision. Please contact me to discuss the revision (also by video chat) if you have questions or comments regarding the revision, or should you need additional time.

When submitting your revised manuscript, please also carefully review the instructions that follow below.

PLEASE NOTE THAT upon resubmission revised manuscripts are subjected to an initial quality control prior to exposition to re-review. Upon failure in the initial quality control, the manuscripts are sent back to the authors, which may lead to delays. Frequent reasons for such a failure are the lack of the data availability section (please see below) and the presence of statistics based on n=2 (the authors are then asked to present scatter plots or provide more data points).

When submitting your revised manuscript, we will require:

1) a .docx formatted version of the final manuscript text (including legends for main figures, EV figures and tables), but without the figures included. Figure legends should be compiled at the end of the manuscript text.

2) individual production quality figure files as .eps, .tif, .jpg (one file per figure), of main figures and EV figures. Please upload these as separate, individual files upon re-submission.

The Expanded View format, which will be displayed in the main HTML of the paper in a collapsible format, has replaced the Supplementary information. You can submit up to 6 images as Expanded View. Please follow the nomenclature Figure EV1, Figure EV2 etc. The figure legend for these should be included in the main manuscript document file in a section called Expanded View Figure Legends after the main Figure Legends section. Additional Supplementary material should be supplied as a single pdf file labeled Appendix. The Appendix should have page numbers and needs to include a table of content on the first page (with page numbers) and legends for all content. Please follow the nomenclature Appendix Figure Sx, Appendix Table Sx etc. throughout the text, and also label the figures and tables according to this nomenclature.

For more details, please refer to our guide to authors:
http://www.embopress.org/page/journal/14693178/authorguide#manuscriptpreparation

Please consult our guide for figure preparation:
http://wol-prod-cdn.literatumonline.com/pb-assets/embo-site/EMBOPress_Figure_Guidelines_061115-1561436025777.pdf

See also the guidelines for figure legend preparation:
https://www.embopress.org/page/journal/14693178/authorguide#figureformat

3) a .docx formatted letter INCLUDING the reviewers' reports and your detailed point-by-point responses to their comments. As part of the EMBO Press transparent editorial process, the point-by-point response is part of the Review Process File (RPF), which will be published alongside your paper.

4) a complete author checklist, which you can download from our author guidelines (https://www.embopress.org/page/journal/14693178/authorguide). Please insert page numbers in the checklist to indicate where the requested information can be found in the manuscript. The completed author checklist will also be part of the RPF.

Please also follow our guidelines for the use of living organisms, and the respective reporting guidelines:
http://www.embopress.org/page/journal/14693178/authorguide#livingorganisms

5) that primary datasets produced in this study (e.g. RNA-seq, ChIP-seq, structural and array data) are deposited in an appropriate public database. If no primary datasets have been deposited, please also state this in a dedicated section (e.g. 'No

primary datasets have been generated and deposited'), see below.

See also: http://embor.embopress.org/authorguide#datadeposition

Please remember to provide a reviewer password if the datasets are not yet public.

The accession numbers and database should be listed in a formal "Data Availability" section that follows the model below. This is now mandatory (like the COI statement). Please note that the Data Availability Section is restricted to new primary data that are part of this study. This section is mandatory. As indicated above, if no primary datasets have been deposited, please state this in this section

Data availability

The datasets produced in this study are available in the following databases:

- RNA-Seq data: Gene Expression Omnibus GSE46843 (https://www.ncbi.nlm.nih.gov/geo/query/acc.cgi?acc=GSE46843)
- [data type]: [name of the resource] [accession number/identifier/doi] ([URL or identifiers.org/DATABASE:ACCESSION])

*** Note - All links should resolve to a page where the data can be accessed. ***

Moreover, I have these editorial requests:

6) We now request the publication of original source data with the aim of making primary data more accessible and transparent to the reader. You will receive a separate email with instructions for providing source data with your revised manuscript, including information how to upload and organize the files.

7) Our journal encourages inclusion of *data citations in the reference list* to directly cite datasets that were re-used and obtained from public databases. Data citations in the article text are distinct from normal bibliographical citations and should directly link to the database records from which the data can be accessed. In the main text, data citations are formatted as follows: "Data ref: Smith et al, 2001" or "Data ref: NCBI Sequence Read Archive PRJNA342805, 2017". In the Reference list, data citations must be labeled with "[DATASET]". A data reference must provide the database name, accession number/identifiers and a resolvable link to the landing page from which the data can be accessed at the end of the reference. Further instructions are available at: http://www.embopress.org/page/journal/14693178/authorguide#referencesformat

8) Regarding data quantification and statistics, please make sure that the number "n" for how many independent experiments were performed, their nature (biological versus technical replicates), the bars and error bars (e.g. SEM, SD) and the test used to calculate p-values is indicated in the respective figure legends (also for EV and Appendix figures). Please also check that all the p-values are explained in the legend, and that these fit to those shown in the figure. Please provide statistical testing where applicable. Please avoid the phrase 'independent experiment' but clearly state if these were biological or technical replicates. Please also indicate (e.g. with n.s.) if testing was performed, but the differences are not significant. In case n=2, please show the data as separate datapoints without error bars and statistics. See also: http://www.embopress.org/page/journal/14693178/authorguide#statisticalanalysis

If n<5, please show single datapoints for diagrams.

9) Please add scale bars of similar style and thickness to all microscopic images, using clearly visible black or white bars (depending on the background). Please place these in the lower right corner of the images themselves. Please do not write on or near the bars in the image but define the size in the respective figure legend.

10) Please also note our reference format: http://www.embopress.org/page/journal/14693178/authorguide#referencesformat

11) We updated our journal's competing interests policy in January 2022 and request authors to consider both actual and perceived competing interests. Please review the policy https://www.embopress.org/competing-interests and update your competing interests if necessary. Please name this section 'Disclosure and Competing Interests Statement' and put it after the Acknowledgements section.

12) We now use CRediT to specify the contributions of each author in the journal submission system. CRediT replaces the author contribution section. Please use the free text box to provide more detailed descriptions and do NOT provide your final manuscript text file with an author contributions section. See also our guide to authors: https://www.embopress.org/page/journal/14693178/authorguide#authorshipguidelines

13) All Materials and Methods need to be described in the main text using our 'Structured Methods' format, which is required for all research articles. According to this format, the Methods section should include a Reagents and Tools Table (listing key

reagents, experimental models, software, and relevant equipment and including their sources and relevant identifiers), uploaded as separate file, and a Methods section in which we encourage the authors to describe their methods using a step-by-step protocol format with bullet points, to facilitate the adoption of the methodologies across labs. More information on how to adhere to this format as well as downloadable templates (.doc) for the Reagents and Tools Table can be found in our author guidelines (section 'Structured Methods'):

https://www.embopress.org/page/journal/14693178/authorguide#manuscriptpreparation

14) Please provide a title with not more than 100 characters (including spaces) and without the colon.

15) Please add up to 5 keywords to the manuscript and order the manuscript sections like this, using only these names:
Title page - Abstract (max. 175 words) - Keywords - Introduction - Results - Discussion - Methods - Data availability section - Acknowledgements (please include here all the funding information) - Disclosure and Competing Interests Statement - References - Figure legends - Expanded View Figure legends

16) Please make sure that all the funding information is also entered into the online submission system and that it is complete and similar to the one in the acknowledgement section of the manuscript text file.

We would also welcome the submission of cover suggestions, or motifs to be used by our Graphics Illustrator in designing a cover.

As part of the EMBO publication's Transparent Editorial Process, EMBO reports publishes online a Review Process File (RPF) to accompany accepted manuscripts. This File will be published in conjunction with your paper and will include the referee reports, your point-by-point response and all pertinent correspondence relating to the manuscript.

You are able to opt out of this by letting the editorial office know (emboreports@embo.org). If you do opt out, the Review Process File link will point to the following statement: "No Review Process File is available with this article, as the authors have chosen not to make the review process public in this case."

I look forward to seeing a revised form of your manuscript when it is ready.

Yours sincerely,

Achim Breiling
Senior Editor
EMBO Reports

Referee #1:

In their manuscript, "Antibody mediated protection against malaria sporozoite infection: relevance of the Fc fragment and epitope valency, Flores-Garcia and colleagues explore the role of the Fc region of antibodies that bind to the NPNA region of P. falciparum circumsporozoite protein. To do this, investigators generated Fab fragments and also made antibody mutants with sequences known to reduce Fc functions. In order to evaluate the efficacy of each of these antibodies in blocking infection, then used a transgenic P. berghei parasite that expresses PfCSP with different number of repeats in the sequence of CSP. Their data are consistent with the model that Fc functions play a minor role in neutralization. Taken together, these data provide an important insight into the mechanism of neutralization for antibodies directed at the repeat region of CSP.

Specific points:
1. There are several points throughout the manuscript where the literature needs to be better cited. For example, line 60 and line 249. In general, a more comprehensive assessment of the current state of the literature and why others have suggested that complement plays a role in blocking parasites would be helpful.

2. In the discussion, it would be useful to more comprehensively discuss the limitations of this study - do the authors think their findings apply to all different CSP repeats, or just to NPNA. Do they think all different CSP antibodies have these same properties, or just those generated in their studies?
3. Assessment of RTS,S trials is an oversimplification, as protection has varied dramatically. Line 60 should be modified to reflect this reality.
4. In the abstract, authors suggest "1 or 2" NPNA3s are required for protection, but there is a clear difference between Figure 7 C and D. It seems that multiple (2) NPNA3s is required, but not 1. This should be reflected in the abstract and throughout the text.
5. The data shown in Figure 3 is very interesting and suggestive of different cell biological properties elicited by different types of antibodies. Difference in secretion between anti-CSP bound sporozoites is also observed in the P. yoelii model (PMID:

34348141). Which of these features do the authors think are contributing the neutralization? Has this been observed elsewhere? It would be interesting to help the reader better understand this phenomenon.

Referee #2:

This study explores the role of the protective efficacy of antibodies against CSP and in particular the role of the Fc portion of antibodies in this neutralisation. The authors investigate this by comparing the full Mab with its corresponding Fab fragment. In addition they examine the influence of multiple antibody binding in protection by using recombinant CSP with differing NANP repeats.

This is a careful study, and the experiments appear well conducted and thoughtfully analysed. The primary finding is that the Fc portion is not necessary since both Fabs and Mabs have the same neutralisation capacity. Consistent with this, mutations in the Fc moiety did not affect efficacy. The EM studies were interesting in that they confirm that Fabs do not induce CSP shedding but also revealed significant ultrastructural changes underneath the membrane of affected sporozoites.

The observation that Fabs have direct anti parasite effects but do not kill sporozoites in suspension is consistent with previous studies that show that although parasite-dependent antibody cytotoxicity is important for neutralizing sporozoites during migration through the skin, abrogation of liver infection is driven by a non-cytotoxic mechanism.

This study nicely complements the existing literature, except for the observation that the number of (NPNA) repeats is not necessarily critical for protection which appears a novel finding.

Some suggestions for improvement:
1. Given the finding of the number of repeats is not necessary for protection it would be important to explicitly verify that the expected numbers of NANP repeats are being expressed on the parasites. This could be done either by PCR/sequencing from parasites or western blotting of parasite material and visualising a size shift. Perhaps this has been mentioned in the report but I could not see this.
2. Figures 1 and 2 - Antibody concentrations should be presented in molar units rather than mass (µg/mL) to allow more accurate comparison and interpretation of antibody efficacy.
3. Line 154 - Please include the reference citation instead of just the numerical placeholder for clarity.
4. Liver burden results - Include representative IVIS images showing liver bioluminescence to enhance data transparency and visual clarity of infection outcomes.

Referee #3:

Overall Assessment
The manuscript by Zavala et al. provides important mechanistic insights into antibody-mediated protection against Plasmodium falciparum sporozoite infection, focusing on the relative contributions of Fc effector functions and epitope valency of the circumsporozoite protein (CSP).

Using human monoclonal antibodies (mAbs) specific for the conserved (NPNA)3 repeat epitopes, the authors convincingly demonstrate that Fc-dependent effector functions are not essential for neutralization. Fab fragments retained full antigen-binding capacity and inhibited sporozoite motility and liver infection comparably to intact IgG, while Fc-mutated antibodies (LALA and KA variants) conferred protection equivalent to wild-type antibodies. These findings argue against a critical role for Fc-mediated effector mechanisms in the early stages of sporozoite neutralization, where rapid hepatocyte invasion likely precludes significant involvement of innate immune cells or complement activation.

The study further shows that antibody-induced CSP shedding and direct parasite killing in suspension, although observed with intact IgG, are not required for protection, as Fab fragments lacking these activities still neutralized infection. To dissect antigen valency, the authors generated transgenic P. berghei lines expressing CSP molecules with varying numbers of (NPNA)3 epitopes. Neutralization efficacy in intravenous challenge models was comparable across constructs, indicating that monovalent binding to a single epitope suffices to block liver infection.

However, in mosquito bite challenges, sterile protection was enhanced when CSP expressed two separate epitopes, correlating with increased cytotoxicity in a 3D killing assay. These results suggest that while monovalent binding suffices during blood-stage transit, bivalent binding enhances cytotoxic activity in the skin. Collectively, the data challenge the prevailing notion that Fc effector functions and extensive Fab-Fab interactions are indispensable, instead highlighting the sufficiency of direct antigen binding and the added benefit of epitope multiplicity in natural transmission contexts.

The work has significant implications for malaria vaccine design and monoclonal antibody development, underscoring that

protective efficacy can be achieved through antibodies targeting even a single conserved CSP epitope, while optimization of epitope presentation may further enhance sterile immunity. Although Fc functions may not be critical for immediate neutralization, their role in antibody persistence, tissue distribution, and adaptive immunity remains relevant for long-term protection strategies. This study thus advances our understanding of the minimal structural requirements for antibody-mediated protection and provides a rational framework for next-generation immunoprophylactic interventions.

Strengths
• Clear experimental rationale and logical progression from Fab/IgG comparisons to Fc mutants and epitope valency.
• Integration of structural and functional assays, providing a multidimensional view of antibody activity.
• Use of multiple challenge models, including mosquito bite assays, which add biological relevance.
• Informative figures that align structural and functional findings.
• Valuable contribution to translational understanding of antibody requirements for malaria prevention.

Limitations
• Dose normalization: Fab and IgG were compared on a mass basis rather than molar or binding-site equivalence. For example, 8 µg of Fab corresponds to ~12 µg of IgG when accounting for molecular weight and valency. This could confound direct functional comparisons. A supplemental figure overlaying IgG and Fab based on molarity and corrected binding sites would clarify potential differences.
• Single antibody clone: The study relies heavily on mAb317, isolated from an RTS,S vaccine recipient, inherently biased toward NANP repeats. This restricts conclusions to vaccine-induced responses and may not reflect the broader repertoire elicited by natural infection.
• Challenge models: Intravenous sporozoite injection bypasses the skin stage of infection, and mosquito bite challenges were limited in scope.
• Posttranslational modifications: The study did not examine Fc glycosylation (e.g., N-linked glycan at Asn297) or other PTMs of Fab/IgG (oxidation, deamidation, glycation). Conclusions about Fc independence are based on genetic Fc mutations and Fab comparisons, not natural glycoform variability.
• Comparative context: The authors should discuss why their findings differ from Stefanutti et al. (PMID 40698943) and Wang et al. (PMID 39198635), which reported more prominent roles for Fc functions.
• Effector assays: Complement deposition and FcγR activation were inferred from Fc mutations but not directly measured.
• Epitope valency models: Engineered P. berghei CSP constructs may not fully mimic P. falciparum CSP organization.

Recommendations for Authors
• Normalize Fab vs IgG dosing on a molar or binding-site equivalent basis; consider a supplemental figure overlaying corrected doses. Similar to Supplemental Figure 1 but taking binding sites and molarity into account.
• Expand antibody diversity beyond mAb317 to reduce clone-specific bias. This one might be too much to ask for as it would require substantial experiments.
• Explicitly acknowledge NANP epitope bias and consider inclusion of junctional or C-terminal antibodies.
• Increase reliance on mosquito bite challenges or models that capture the skin stage of infection.
• Incorporate or discuss Fc glycosylation and other PTMs to strengthen translational relevance.
• Complement Fc mutant analyses with direct assays of complement deposition and FcγR activation.
• Discuss differences with Stefanutti et al. and Wang et al. to contextualize findings.
• Streamline methodological detail and ensure consistent figure callouts for clarity.

Conclusion:
This manuscript is clearly written, logically structured, and provides valuable mechanistic insights into antibody-mediated protection against malaria sporozoites. The findings refine our understanding of the minimal structural requirements for protective antibodies and have direct implications for vaccine and monoclonal antibody design. Addressing the outlined limitations-particularly dose normalization, clone diversity, epitope bias, and PTM analysis-would enhance both the rigor and translational impact of the study.

Answer to the critiques and suggestions raised by the reviewers,

We thank the reviewers for their thoughtful comments and suggestions. We hope this manuscript is now acceptable for publication

Reviewer #1.

1. There are several points throughout the manuscript where the literature needs to be better cited. For example, line 60 and line 249. In general, a more comprehensive assessment of the current state of the literature and why others have suggested that complement plays a role in blocking parasites would be helpful.

a) For line 60 in introduction, we have expanded significantly the discussion regarding the results of vaccine trials and added the respective references: page 3, lines 57-68. (PMID: 25913272, PMID: 26342424, PMID: 37883572). We agree with the reviewer that this is important however, since this is not a vaccine manuscript, we are hesitant to further expend these aspects.

b) For line 249 we added a number of references regarding the role of anti-CSP antibodies in protection in animal models and human trial page 9, lines 261-263), (PMID: 19569965, PMID: 25913272, PMID: 26342424, PMID: 29138320)

c) We also discuss why complement has been proposed as an effector mechanism of antibody mediated protection and discuss additional studies that appear to disagree regarding the possible role of the complement system as effector mechanism: page 4, lines 92-99 (PMID: 32718991, PMID:39127054, PMID: 1426138, PMID: 37859300)

2. In the discussion, it would be useful to more comprehensively discuss the limitations of this study - do the authors think their findings apply to all different CSP repeats, or just to NPNA. Do they think all different CSP antibodies have the same properties, or just those generated in their studies?

This matter is now discussed extensively in the discussion section, pages 11-12, lines 347-362, and have added the respective references PMID: 26342424, PMID: 29138320, PMID: 29554083, PMID: 32946741, PMID: 40267218, PMID: 34748617, PMID: 40394009, PMID: 35344575.

3.Assessment of RTS,S trials is an oversimplification, as protection has varied dramatically. Line 60 should be modified to reflect this reality.

We included additional information (as answered to item 1. Page 3, lines 57-68

4. In the abstract, authors suggest "1 or 2" NPNA3s are required for protection, but there is a clear difference between Figure 7 C and D. It seems that multiple (2) NPNA3s is required, but not 1. This should be reflected in the abstract and throughout the text.

The reviewer is correct, and the abstract has been modified accordingly. This is also made clear in the result sections (Page 8 lines 239-242) and discussion (Page12 lines 366-369)

5. The data shown in Figure 3 is very interesting and suggestive of different cell biological properties elicited by different types of antibodies. Difference in secretion between anti-CSP bound sporozoites is also observed in the P. yoelii model (PMID: 34348141). Which of these features do the authors think are contributing the neutralization? Has this been observed elsewhere? It would be interesting to help the reader better understand this phenomenon.

The point raised by the reviewer is interesting, however, that seems to apply to antibodies with different epitope specificity. In our case the specificity of the antibodies is the same and may only differ in being bivalent or monovalent. As the situation described in the above-mentioned paper appears to be different to our observation we would prefer not to comment on this matter

Reviewer #2:

1.Given the finding of the number of repeats is not necessary for protection it would be important to explicitly verify that the expected numbers of NANP repeats are being expressed on the parasites. This could be done either by PCR/sequencing from parasites or western blotting of parasite material and visualizing a size shift. Perhaps this has been mentioned in the report but I could not see this.

We apologize for not adding this important information: All parasites were sequenced after PCR of the transgene using primers that amplified the entire 800-900 bp of the inserted modified CSP gene. This is now described in methods section, page 15, lines 448-450. In addition, Suppl Fig 3B shows size shifts from PCR amplified transgene.

2. Figures 1 and 2 - Antibody concentrations should be presented in molar units rather than mass (μg/mL) to allow more accurate comparison and interpretation of antibody efficacy.

We now show the results of a new experiment in Figure 1 using equimolar amounts of IgG and Fab, the graphs have been modified accordingly.

3. Line 154 - Please include the reference citation instead of just the numerical placeholder for clarity.

This reference is now cited correctly.

4. Liver burden results - Include representative IVIS images showing liver bioluminescence to enhance data transparency and visual clarity of infection outcomes.

We now include an addition to figure 1, showing images of liver infection.

Reviewer #3

Recommendations for Authors

a) Normalize Fab vs IgG dosing on a molar or binding-site equivalent basis; consider a supplemental figure overlaying corrected doses. Similar to Supplemental Figure 1 but taking binding sites and molarity into account.

We now show the results of a new experiment in Figure 1 using equimolar amounts of IgG and Fab, the graphs have been modified accordingly.

b) Expand antibody diversity beyond mAb317 to reduce clone-specific bias. This one might be too much to ask for as it would require substantial experiments.

We report data using two different mAbs: mAb317 (Fig 1B) and mAb311 (Fig 1C). As stated in the text, mAb 311 belongs to a different VH family and has similar epitope specificity to mAb317 (page 5, lines 140-144).

c) Explicitly acknowledge NANP epitope bias and consider inclusion of junctional or C-terminal antibodies.

In the discussion section we acknowledge the epitope bias and explain the reasons why we chose to analyze these antibodies. We also added comments on Fc functions and homotypic interaction regarding mAbs that recognize junction, minor repeat and C-term epitopes. Page 11-12, lines 347-362.

d) Increase reliance on mosquito bite challenges or models that capture the skin stage of infection.

In the section regarding epitope valency, we report detailed protection data obtained by both mosquito bites and iv. injections (Figs 6 and 7) as we believe both assays provide valuable information about potentially different mechanisms of protection. However, when assessing protection by Fab fragment vs IgG the protective effect of Fab needs to be done by injecting

simultaneously the parasites and the Fab, as these fragments are rapidly cleared from circulation (as stated in page5, lines 132-136). This is a major limitation for mosquito bites experiments as this challenge may take as long as 30 minutes after injection of antibody, a time during which most of the Fab has cleared from circulation.

e) Incorporate or discuss Fc glycosylation and other PTMs to strengthen translational relevance.

We agree that this is a topic of importance that we neglected to discuss in the original manuscript. We have now added a paragraph in the discussion section describing the importance of Fc glycosylation on antibody effector functions Page 10-11, lines 308-318.

f) Complement Fc mutant analyses with direct assays of complement deposition and FcγR activation

We focused our studies on the in vivo effect of Fc fragments. There are several reports describing the in vitro activation of complement cascade by antibodies (and sporozoites (PMID: 32718991, PMID:39127054), as well as the induction of in vitro ADCC (PMID: 32718991) These in vitro results are not disputed; our studies try to determine the relevance of these mechanisms in the in vivo effect of antibodies.

a) *We believe there are additional complexities regarding the in vitro studies, while antibodies enhance complement activation the deposition of complement components can also happen by parasite activation of the alternative pathway, and this may not affect in vivo parasite infectivity (*PMID: 1426138, PMID: 37859300). *Moreover, C1q -which initiates the classical pathway- can also bind to C3b generated by alternative activation (PMID 2958692). There are no sensitive assays that could distinguish -at least in the sporozoites surface- which complement components are generated by the classical or alternative activation pathways. Therefore, we believe there are too many confounders in these in vitro experiments, and the results are difficult to interpret. We added comments on the role of complement sporozoite infection page 4, lines 92-99.*
b) *We have added new data (Suppl Fig. 2) showing the binding of the Fc mutated antibodies to human and mouse FcgR1. The results are in agreement with what is described in the literature for these Fc mutations: LALA antibodies have a severely diminished binding to the Fc receptor while KA antibodies bind normally.*

g) Discuss differences with Stefanutti et al. and Wang et al. to contextualize findings.

In the discussion section, page 10, lines 282-292 we added a new paragraph discussing our findings and what is described in the mentioned papers.

h) Streamline methodological detail and ensure consistent figure callouts for clarity.

We hope we have now improved the presentation of the manuscript.

Dear Dr. Zavala,

Thank you for the submission of your revised manuscript to our editorial offices. I have now received the reports from the two referees that I asked to re-evaluate the study, you will find below. As you will see, the referees now fully support the publication of your study in EMBO reports.

Before we can proceed with formal acceptance, I have the editorial requests below I ask you to address in a final revised manuscript. Please also provide a final p-b-p-response to the editorial requests.

Editorial requests:

- Please provide a final title for your manuscript without colon and with not more than 100 characters including spaces.

- Is author affiliation 4 (PATH) a company? If yes, please indicate this and comment on this in the Disclosure and Competing Interests Statement.

- Please provide the abstract written in present tense throughout.

- Please fuse the main figures to have not more than 6. Then, please update all callouts. The mouse images in 2B are very small. Please provide these larger, maybe as a separate panel.

- The Expanded View format, which will be displayed in the main HTML of the paper in a collapsible format, has replaced the Supplementary information. Thus, please combine the figures presently shown in the Appendix to have not more than 5 EV figures (I think the data would fit into 2 EV figures) and upload these as separate EV figures. Please follow the nomenclature Figure EV1, Figure EV2 etc. and put the figure legend for these in the main manuscript document file in a section called Expanded View Figure Legends after the main Figure Legends section (see below). Please update all callouts.

- Please add the information shown in Appendix Table S1 to the Reagents & Tools table (section oligonucleotides). Please update all callouts (pointing to the Reagents & Tools table) and finally delete the Appendix file.

- Please add the primer sequences directly into the Reagents & Tools Table (not as separate table below). Moreover, please remove the instruction text.

- Please add up to five keywords to the manuscript and order the manuscript sections like this, using only these names:
Title page - Abstract - Keywords - Introduction - Results - Discussion - Methods - Data availability section - Acknowledgements - Disclosure and Competing Interests Statement - References - Figure legends - Expanded View Figure legends

- Please check again that the number "n" for how many independent experiments were performed, their nature (biological versus technical replicates), the bars and error bars (e.g. SEM, SD) and the test used to calculate p-values is indicated in the respective figure legends (main and EV figures). Please also check that all the p-values are explained in the legend, and that these fit to those shown in the figure. Please provide statistical testing where applicable. Please avoid the phrase 'independent experiment' but clearly state if these were biological or technical replicates. Please also indicate (e.g. with n.s.) if testing was performed, but the differences are not significant. In case n=2, please show the data as separate datapoints without error bars and statistics. See also:

https://link.springer.com/journal/44319/submission-guidelines#cms-Figure-and-data-presentation

If n<5, please show single datapoints for diagrams. For some panels (e.g. Fig. S1) , n is not indicated. Could error bars be added to the bar diagrams shown in Figs. 1A, 1B, 2B, 6 and S4? Moreover:
- Please note that the exact p values are not provided in the legend of figure 8A
- Please note that information related to n is missing in the legend of figure 8D
- Please note that the error bars are not defined in the legends of figures 6A-E; 8A-D; S3 A.
- Please note that the scale bar needs to be defined for figure 4C

- Please add scale bars of similar style and thickness to all microscopic images, using clearly visible black or white bars (depending on the background). Please place these in the lower right corner of the images themselves. Please do not write on or near the bars in the image but define the size in the respective figure legend. Presently, some scale bars have text nearby.

- Please make sure that each final figure panel is called out separately and that the panels are called out sequentially. Presently, it seems there are no separate callouts for panels 2A and 4C. Please check.

- Please make sure that all the funding information is also entered into the online submission system and that it is complete and

similar to the one in the acknowledgement section of the manuscript text file. It appears that the following items are missing, but there might be more that need to be listed in the submission system: the French Government's Investissement d'Avenir program, Laboratoire d'Excellence "Integrative Biology of Emerging Infectious Diseases" - project number ANR-10-LABX-62-IBEID. Please check.

- Please use our reference format:
https://link.springer.com/journal/44319/submission-guidelines#cms-Reference-guidelines

- Please provide a complete author checklist.

- Thanks for providing the source data. Please upload the source data as one folder per final main figure, grouping together the separate files (in excel format) for the panels, and ZIPed together. Please clearly label these files, one file per panel, and indicate in the checklist for which panel SD was provided. Presently, source data for 8D appears to be missing. To what figure(s) is the SD labelled 'A_B (numerical data)' belonging to? Where is the SD for Figure 4? Please check.

In addition, I would need from you uploaded separately:
- a short, two-sentence summary of the manuscript (not more than 35 words).
- two to four short (!) bullet points highlighting the key findings of your study (two lines each).
- a schematic summary figure as separate file that provides a sketch of the major findings (not a data image) in jpeg or tiff format (with the exact width of 550 pixels and a height of exactly 300 pixels) that can be used as a visual synopsis on our website.

I look forward to seeing the further revised version of your manuscript when it is ready. Please let me know if you have questions regarding the revision.

Best,

Achim Breiling
Senior Editor
EMBO Reports

Referee #1:

The authors have addressed all relevant points made in the reviews.

Referee #3:

The authors addressed all concerns this reviewer had and improved their manuscript significantly.

The authors have addressed all minor editorial requests.

Fidel Zavala
Johns Hopkins Bloomberg School of Public Health
Molecular Microbiology and Immunology
United States

Dear Dr. Zavala,

Thank you for the submission of your final revised manuscript to our editorial offices. I now went through it and your final p-b-p-response and consider the remaining editorial requests as adequately addressed.

I am thus very pleased to accept your manuscript for publication in the next available issue of EMBO reports. Thank you for your contribution to our journal.

Your manuscript will be processed for publication by EMBO Press. It will be copy edited and you will receive page proofs prior to publication. Please note that you will be contacted by Springer Nature Author Services to complete licensing and payment information.

You may qualify for financial assistance for your publication charges - either via a Springer Nature fully open access agreement or an EMBO initiative. Check your eligibility: https://link.springer.com/journal/44319/how-to-publish-with-us

Should you be planning a Press Release on your article, please get in contact with embo_production@springernature.com as early as possible in order to coordinate publication and release dates.

If you have any questions, please do not hesitate to contact the Editorial Office. Thank you for your contribution to EMBO Reports.

Yours sincerely,

Achim Breiling
Senior Editor
EMBO Reports

>>> Please note that it is EMBO Reports policy for the transcript of the editorial process (containing referee reports and your response letter) to be published as an online supplement to each paper. If you do NOT want this, you will need to inform the Editorial Office via email immediately. More information is available here: https://link.springer.com/partners/embo-press/editorial-policies#Peer%20review

EMBO Press Author Checklist

Corresponding Author Name: Fidel Zavala	
Journal Submitted to: EMBO Reports	
Manuscript Number: EMBOR-2025-62918-T	

USEFUL LINKS FOR COMPLETING THIS FORM
The EMBO Journal - Author Guidelines
EMBO Reports - Author Guidelines
Molecular Systems Biology - Author Guidelines
EMBO Molecular Medicine - Author Guidelines

Reporting Checklist for Life Science Articles (updated January
This checklist is adapted from Materials Design Analysis Reporting (MDAR) Checklist for Authors. MDAR establishes a minimum set of requirements in transparent reporting in the life sciences (see Statement of Task: 10.31222/osf.io/9sm4x). Please follow the journal's guidelines in preparing your
Please note that a copy of this checklist will be published alongside your article.

Abridged guidelines for figures
1. Data
The data shown in figures should satisfy the following conditions:
- the data were obtained and processed according to the field's best practice and are presented to reflect the results of the experiments in an accurate and unbiased manner.
- ideally, figure panels should include only measurements that are directly comparable to each other and obtained with the same assay.
- plots include clearly labeled error bars for independent experiments and sample sizes. Unless justified, error bars should not be shown for technical
- if n<5, the individual data points from each experiment should be plotted. Any statistical test employed should be justified.
- Source Data should be included to report the data underlying figures according to the guidelines set out in the authorship guidelines on Data

2. Captions
Each figure caption should contain the following information, for each panel where they are relevant:
- a specification of the experimental system investigated (eg cell line, species name).
- the assay(s) and method(s) used to carry out the reported observations and measurements.
- an explicit mention of the biological and chemical entity(ies) that are being measured.
- an explicit mention of the biological and chemical entity(ies) that are altered/varied/perturbed in a controlled manner.
- the exact sample size (n) for each experimental group/condition, given as a number, not a range;
- a description of the sample collection allowing the reader to understand whether the samples represent technical or biological replicates (including how many animals, litters, cultures, etc.).
- a statement of how many times the experiment shown was independently replicated in the laboratory.
- definitions of statistical methods and measures:
  - common tests, such as t-test (please specify whether paired vs. unpaired), simple χ2 tests, Wilcoxon and Mann-Whitney tests, can be unambiguously identified by name only, but more complex techniques should be described in the methods section;
  - are tests one-sided or two-sided?
  - are there adjustments for multiple comparisons?
  - exact statistical test results, e.g., P values = x but not P values < x;
  - definition of 'center values' as median or average;
  - definition of error bars as s.d. or s.e.m.

Please complete ALL of the questions below.
Select "Not Applicable" only when the requested information is not relevant for your study.

Materials

Newly Created Materials	Information included in the manuscript?	In which section is the information available? (Reagents and Tools Table, Materials and Methods, Figures, Data Availability Section)
New materials and reagents need to be available; do any restrictions apply?	Yes	All materials and reagents described in this paper are available upon request and under collaborative agrement

Antibodies	Information included in the manuscript?	In which section is the information available? (Reagents and Tools Table, Materials and Methods, Figures, Data Availability Section)
For **antibodies** provide the following information:<br>- Commercial antibodies: RRID (if possible) or supplier name, catalogue number and or/clone number<br>- Non-commercial: RRID or citation	Not Applicable	Antibodies used in these work are not comercial. Oyen D, ot al, PNAS, 2017

DNA and RNA sequences	Information included in the manuscript?	In which section is the information available? (Reagents and Tools Table, Materials and Methods, Figures, Data Availability Section)
**Short novel DNA or RNA including primers, probes**: provide the sequences.	Yes	Primers to amplify transgenes expressed by the parasite are described in the material and methods section and also in reagents and tools file, page 15.

Cell materials	Information included in the manuscript?	In which section is the information available? (Reagents and Tools Table, Materials and Methods, Figures, Data Availability Section)
**Cell lines:** Provide species information, strain. Provide accession number in repository **OR** supplier name, catalog number, clone number, and/**OR** RRID.	Not Applicable	
**Primary cultures:** Provide species, strain, sex of origin, genetic modification status.	Not Applicable	
Report if the cell lines were recently **authenticated** (e.g., by STR profiling) and tested for mycoplasma contamination.	Not Applicable	

Experimental animals	Information included in the manuscript?	In which section is the information available? (Reagents and Tools Table, Materials and Methods, Figures, Data Availability Section)
**Laboratory animals or Model organisms:** Provide species, strain, sex, age, genetic modification status. Provide accession number in repository **OR** supplier name, catalog number, clone number, **OR** RRID.	Yes	Animals used in this study and the murine plasmodium species are described in material and methods section, pages 14-15.
**Animal observed in or captured from the field:** Provide species, sex, and age where possible.	Not Applicable	
Please detail **housing and husbandry conditions**.	Yes	Animals were maintained under controlled environmental conditions, including 40–60% relative humidity, ambient

Plants and microbes	Information included in the manuscript?	In which section is the information available? (Reagents and Tools Table, Materials and Methods, Figures, Data Availability Section)
**Plants:** provide species and strain, ecotype and cultivar where relevant, unique accession number if available, and source (including location for collected wild specimens).	Not Applicable	
**Microbes:** provide species and strain, unique accession number if available, and source.	Yes	Plasmodium berghei, from ATCC, described in methods, page 14.

Human research participants	Information included in the manuscript?	In which section is the information available? (Reagents and Tools Table, Materials and Methods, Figures, Data Availability Section)
If collected and within the bounds of privacy constraints report on age, sex and gender or ethnicity for all study participants.	Not Applicable	

Core facilities	Information included in the manuscript?	In which section is the information available? (Reagents and Tools Table, Materials and Methods, Figures, Data Availability Section)
If your work benefited from core facilities, was their service mentioned in the acknowledgments section?	Yes	Information available in acknowledgments, page 17-18.

Design

Study protocol	Information included in the manuscript?	In which section is the information available? (Reagents and Tools Table, Materials and Methods, Figures, Data Availability Section)
If study protocol has been **pre-registered, provide DOI in the manuscript**. For clinical trials, provide the trial registration number **OR** cite DOI.	Not Applicable	
Report the **clinical trial registration number** (at ClinicalTrials.gov or equivalent), where applicable.	Not Applicable	

Laboratory protocol	Information included in the manuscript?	In which section is the information available? (Reagents and Tools Table, Materials and Methods, Figures, Data Availability Section)
Provide DOI OR other citation details if **external detailed step-by-step protocols** are available.	Yes	Experiments were appproved by the Johns Hopkins ACUC, described in material and methods, page 14.

Experimental study design and statistics	Information included in the manuscript?	In which section is the information available? (Reagents and Tools Table, Materials and Methods, Figures, Data Availability Section)
Include a statement about **sample size** estimate even if no statistical methods were used.	Yes	The statistical analysis is identify in the legend of figures and material and methods section
Were any steps taken to minimize the effects of subjective bias when allocating animals/samples to treatment (e.g. **randomization procedure**)? If yes, have they been described?	Not Applicable	Animales were randomly selected and adscribed to the different experimental groups
Include a statement about **blinding** even if no blinding was done.	Yes	Each experimental group was identified by a code known only to the PI but not to the people in charge to perform the experiments.
Describe **inclusion/exclusion criteria** if samples or animals were excluded from the analysis. Were the criteria pre-established?<br><br>If sample or data points were omitted from analysis, report if this was due to attrition or intentional exclusion and provide justification.	Not Applicable	There was no exclusions. No data points were omitted from analysis.
For every figure, are **statistical tests** justified as appropriate? Do the data meet the assumptions of the tests (e.g., normal distribution)? Describe any methods used to assess it. Is there an estimate of variation within each group of data? Is the variance similar between the groups that are being statistically compared?	Yes	The results were analyzed statistically by a non-parametric test: Kruskal-Wallis and Mann Whitney. Normal distribution was not assumed. There is no estimate of variation. The variance is similar between groups.

Sample definition and in-laboratory replication	Information included in the manuscript?	In which section is the information available? (Reagents and Tools Table, Materials and Methods, Figures, Data Availability Section)
In the figure legends: state number of times the experiment was **replicated** in laboratory.	Yes	The number of times a experiment was replicated is estated in the figure legends.
In the figure legends: define whether data describe **technical or biological replicates**.	Yes	Yes, each figure legend described how the results were obtained and the times performed.

Ethics

Ethics	Information included in the manuscript?	In which section is the information available? (Reagents and Tools Table, Materials and Methods, Figures, Data Availability Section)
Studies involving **human participants**: State details of **authority granting ethics approval** (IRB or equivalent committee(s), provide reference number for approval.	Not Applicable	
Studies involving **human participants**: Include a statement confirming that **informed consent** was obtained from all subjects and that the experiments conformed to the principles set out in the WMA Declaration of Helsinki and the Department of Health and Human Services Belmont Report.	Not Applicable	
Studies involving **human participants:** For publication of **patient photos**, include a statement confirming that consent to publish was obtained.	Not Applicable	
Studies involving experimental **animals**: State details of **authority granting ethics approval** (IRB or equivalent committee(s), provide reference number for approval. Include a statement of compliance with ethical regulations.	Yes	At Johns Hopkins University, every procedure performed was approved by the Animal Care and Use Committee, Protocol #:MO21H417. At the Pasteur Institute: experiments were performed according to French and European regulations and were approved by the Ethics Committee #89
Studies involving **specimen and field samples:** State if relevant **permits** obtained, provide details of authority approving study; if none were required, explain why.	Not Applicable	

Dual Use Research of Concern (DURC)	Information included in the manuscript?	In which section is the information available? (Reagents and Tools Table, Materials and Methods, Figures, Data Availability Section)
Could your study fall under dual use research restrictions? Please check biosecurity documents and list of **select agents and toxins** (CDC): https://www.selectagents.gov/sat/list.htm	Not Applicable	
If you used a select agent, is the security level of the lab appropriate and reported in the manuscript?	Not Applicable	
If a study is subject to dual use research of concern regulations, is the name of the **authority granting approval and reference number** for the regulatory approval provided in the manuscript?	Not Applicable	

Reporting

The MDAR framework recommends adoption of discipline-specific guidelines, established and endorsed through community initiatives. Journals have their own policy about requiring specific guidelines and recommendations to complement MDAR.

Adherence to community standards	Information included in the manuscript?	In which section is the information available? (Reagents and Tools Table, Materials and Methods, Figures, Data Availability Section)
State if relevant guidelines or checklists (e.g., **ICMJE, MIBBI, ARRIVE, PRISMA**) have been followed or provided.	Not Applicable	
For **tumor marker prognostic studies,** we recommend that you follow the **REMARK** reporting guidelines (see link list at top right). See author guidelines, under 'Reporting Guidelines'. Please confirm you have followed these guidelines.	Not Applicable	
For **phase II and III randomized controlled trials**, please refer to the **CONSORT** flow diagram (see link list at top right) and submit the CONSORT checklist (see link list at top right) with your submission. See author guidelines, under 'Reporting Guidelines'. Please confirm you have submitted this list.	Not Applicable	

Data Availability

Data availability	Information included in the manuscript?	In which section is the information available? (Reagents and Tools Table, Materials and Methods, Figures, Data Availability Section)
Have **primary datasets** been deposited according to the journal's guidelines (see 'Data Deposition' section) and the respective accession numbers provided in the Data Availability Section?	Not Applicable	
Were **human clinical and genomic datasets** deposited in a public access-controlled repository in accordance to ethical obligations to the patients and to the applicable consent agreement?	Not Applicable	
Are **computational models** that are central and integral to a study available without restrictions in a machine-readable form? Were the relevant accession numbers or links provided?	Not Applicable	
If publicly available data were reused, provide the respective **data citations in the reference list**.	Not Applicable	
